# Self-locking stand-alone cage versus cage-plate fixation in monosegmental anterior cervical discectomy and fusion with a minimum 2-year follow-up: a systematic review and meta-analysis

**DOI:** 10.1186/s13018-023-03885-4

**Published:** 2023-06-02

**Authors:** Yu Zhang, Jidong Ju, Jinchun Wu

**Affiliations:** Department of Orthopaedics, Jingjiang People’s Hospital, The Seventh Affiliated Hospital of Yangzhou University, Jingjiang, Taizhou, Jiangsu Province, 214500 China

**Keywords:** Cervical spinal fusion, Self-locking stand-alone cage, Single segment, Long-term effectiveness, Meta-analysis

## Abstract

**Background:**

Currently, self-locking stand-alone cages (SSC) are commonly applied in anterior cervical discectomy and fusion (ACDF), as are cage-plate constructs (CPC). However, it remains controversial concerning the long-term effectiveness of both apparatuses. Our purpose is to compare long-term effectiveness of SSC with CPC in monosegmental ACDF.

**Methods:**

Four electronic databases were queried to identify studies comparing SSC versus CPC in monosegmental ACDF. The meta-analysis was carried out with the use of the Stata MP 17.0 software package.

**Results:**

Ten trials with 979 patients were included. Compared to CPC, SSC significantly reduced operative time, intraoperative blood loss, duration of hospitalisation, cervical Cobb angle at final follow-up, 1-month postoperative dysphagia rate, and incidence of adjacent segment degeneration (ASD) at final follow-up. No significant difference was found regarding 1-month postoperative cervical Cobb angle, JOA scores, NDI scores, fusion rate and cage subsidence rate at final follow-up.

**Conclusion:**

Both devices achieved similar long-term effectiveness in monosegmental ACDF regarding JOA scores, NDI scores, fusion rate and cage subsidence rate. SSC had significant advantages over CPC in reducing surgical duration, intraoperative bleeding, duration of hospitalisation, as well as rates of dysphagia and ASD after surgery. Therefore, SSC is a better option than CPC in monosegmental ACDF. However, SSC is inferior to CPC in maintaining cervical curvature at long-term follow-up. Whether radiological changes affect clinical symptoms needs confirmation in trials with longer follow-up.

**Supplementary Information:**

The online version contains supplementary material available at 10.1186/s13018-023-03885-4.

## Introduction

With rapid economic development, changes in lifestyles and increasing work pressure, the number of people suffering from neck pain is increasing continuously. It was reported that approximately 220 million people around the world were affected by neck pain, which brought a huge economic burden to society [1]. Neck pain in adults is usually attributed to degenerative cervical spondylosis (DCS), which in severe cases can develop into spinal cord dysfunction. DCS is a series of clinical syndromes resulting from damage to the spinal cord, nerves and blood vessels caused by degeneration of cervical intervertebral discs and subsequent changes. This debilitating disorder can be treated by conservative means in the early stages. However, once conservative treatment is no longer effective or symptoms continue to worsen, the compressed spinal cord is in danger of irreversible damage. To relieve nerve compression and improve symptoms, surgical treatment should be performed as soon as possible [2].

Currently, anterior cervical discectomy and fusion (ACDF) is widely recognised a gold standard for treating DCS. Through the use of anterior plate fixation, traditional ACDF can provide immediate cervical spinal stability, direct sufficient and effective nerve decompression, normalize intervertebral height, and reconstruct cervical physiological curvature, as well as reducing the incidence of pseudoarthrosis. The placement of a titanium plate can provide additional stability to the operated segment, preventing collapse of the interbody fusion device and the formation of a kyphotic deformity [3]. However, intraoperative placement of the plate requires as much exposure of the surgical field as possible, which is usually accompanied by complications, such as anterior soft tissue injury, postoperative dysphagia and degeneration of adjacent segments [4–6]. Lu et al. attributed postoperative dysphagia to soft tissue oedema at the surgical location, surrounding hematoma, esophageal injury, and tissue adhesion around the plate [7]. Cage-plate construct (CPC) alters the normal biomechanical state of the cervical spine, which leads to loss of motion at the operated segment, concentrated stress loads on adjacent segments, and increased abnormal activity, thereby accelerating adjacent segment degeneration (ASD) [8].

With the intention of reducing the risk of the complications mentioned above, a novel self-locking stand-alone cage (SSC) based on the zero-notch design concept came into being. It was designed to allow self-locking screws to be inserted through the fusion device into the vertebral body of the adjacent segments without plate fixation. The key difference between SSC and CPC is that in the process of operation, there is no need to expose a large anatomical area, which significantly reduces the surgical injury and the formation of postoperative scars. SSCs can effectively avoid invasion of important structures located in the front of the cervical spine, thereby reducing the incidence of postoperative complications such as hematoma, ASD and postoperative dysphagia [9, 10]. Studies have shown that SSC has the similar clinical efficacy compared with cage-plate fixation [8, 11, 12]. It can effectively obtain good stability of fused segments, thereby ensuring eventual bone fusion. Nevertheless, it faces controversy in terms of maintaining the physiological curvature of cervical spine, preventing the subsidence of fusion devices and so on [13, 14].

Some scholars conducted meta-analyses and further compared the efficacy and complications between SSC and CPC in treating DCS, yet the findings remained diverse [15–20]. Limitations existed among these previous meta-analyses, including one fact that some papers involved different surgical segments and different lengths of follow-up. Currently, the superiority of SSC over CPC in terms of short- and medium-term efficacy and safety has been confirmed in numerous meta-analyses. Still, some meta-analysis results are not clear when it comes to comparing the long-term outcomes of SSC versus CPC [15, 17, 18]. One explanation for this phenomenon is that variations in surgical segments and follow-up lengths may influence the results of the meta-analyses. Up to now, no specific meta-analysis has been carried out to investigate the long-term benefits and complications between SSC and CPC in monosegmental ACDF. Therefore, our aim is to evaluate the long-term effectiveness and security of SSC compared to traditional cage-plate fixation in the treatment of monosegmental ACDF with a follow-up length of ≥ 2 years, in attempt to provide convincing evidence for clinicians to make clinical decisions.

## Methods

### Literature search

This study has been registered with PROSPERO (CRD 42022373028). Literature was identified by searching the electronic databases mentioned below: Pubmed, Embase, Web of Science and Cochrane Library. Publication period was from the start-up of the databases to 30 January 2023. The complete search syntax for each database was recorded in the Additional file 1. First, studies were assessed for inclusion based on their titles and abstracts. Subsequently, studies were included based on a review of the full text. In cases where several researches included results from the same source, the most recent study was included. Additionally, citations of articles were searched for the identification of relevant studies that had not been part of the initial literature search. The selection process was carried out by two independent reviewers. Final inclusion was based on the consensus of the two reviewers. These studies were cross-referenced to identify any other relevant studies. If there was any uncertainty, the third author would discuss it with them and make a decision until the final results were reached.

### Study selection

Inclusion criteria: (1) Study population: Patients with definite clinical and imaging diagnosis of degenerative cervical spondylosis necessitating monosegmental anterior cervical surgery. (2) Study design: observational studies (OS) or randomised controlled trials (RCT) that compared the efficacy of SSC versus conventional CPC in monosegmental ACDF. (3) Outcomes: operation time, intraoperative blood loss, length of hospital stay, Japanese orthopaedic association (JOA) score, neck disability index (NDI) score, cervical Cobb angle, fusion rate, incidence of cage subsidence, incidence of postoperative dysphagia, incidence of adjacent segmental degeneration. Studies included at least one of the outcomes listed above. (4) Studies with a postoperative follow-up of at least two years. Exclusion criteria: 1. Study population with multilevel cervical spondylosis or with other cervical disorders, such as fracture, infection, tumour and congenital deformity, or with a previous history of cervical spine surgery. 2. Non-comparative studies. 3. Reduplicated publications, biomechanical studies, cadaveric experiments, animal experiments, reviews, meta-analyses, case reports, letters and conference abstracts. 4. Original data that were incomplete or unavailable for analysis. 5. Non-English literature.

### Data extraction

Data were extracted independently by two reviewers using a standardised form. When differences of judgement arose, they were addressed by means of discussion with a third researcher. From the included literature, we extracted data, including first author, publication date, study type, country, number of cases, age, follow-up time and outcomes.

### Quality assessment

Both researchers independently assessed the quality of the included studies. Randomised controlled trials were evaluated for potential risk of bias according to the Cochrane Collaboration's risk of bias tool. In addition, the Newcastle–Ottawa scale (NOS) was used to assess observational studies. Three categories of bias (selection, comparability and outcome) were assessed and scored by answering eight questions, with a maximum score of 9. High quality was defined as a score of at least 6. Then the results were compared and a third evaluator was involved to reach consensus as required.

### Statistical analysis

The meta-analysis was performed using Stata MP 17.0 (Stata Corporation, College Station, TX, USA). The SSC group was compared with the CPC group. For dichotomous outcomes, effect sizes were estimated as odds ratios (OR) and 95% confidence intervals (CI). For continuous outcomes, effect sizes were expressed as weighted mean differences (WMD) and 95% confidence intervals. The heterogeneity of the included studies was assessed using the I^2^ statistics. I^2^ > 50% indicated heterogeneity among included studies and a random-effects model was used. Otherwise, a fixed-effects model was used for pooling. Different publication years, countries (Chinese or foreign), and study designs (RCTs or observational studies) were considered as possible sources of significant heterogeneity. Meta-regression analysis or subgroup analysis was used to further explore the potential sources of heterogeneity. Sensitivity analysis was conducted by eliminating individual studies one by one to evaluate the robustness of the pooled results. Publication bias was assessed with the Egger's test. Statistical significance was interpreted as *p*-value < 0.05.

## Results

### Search results and study characteristics

**Fig. 1:**
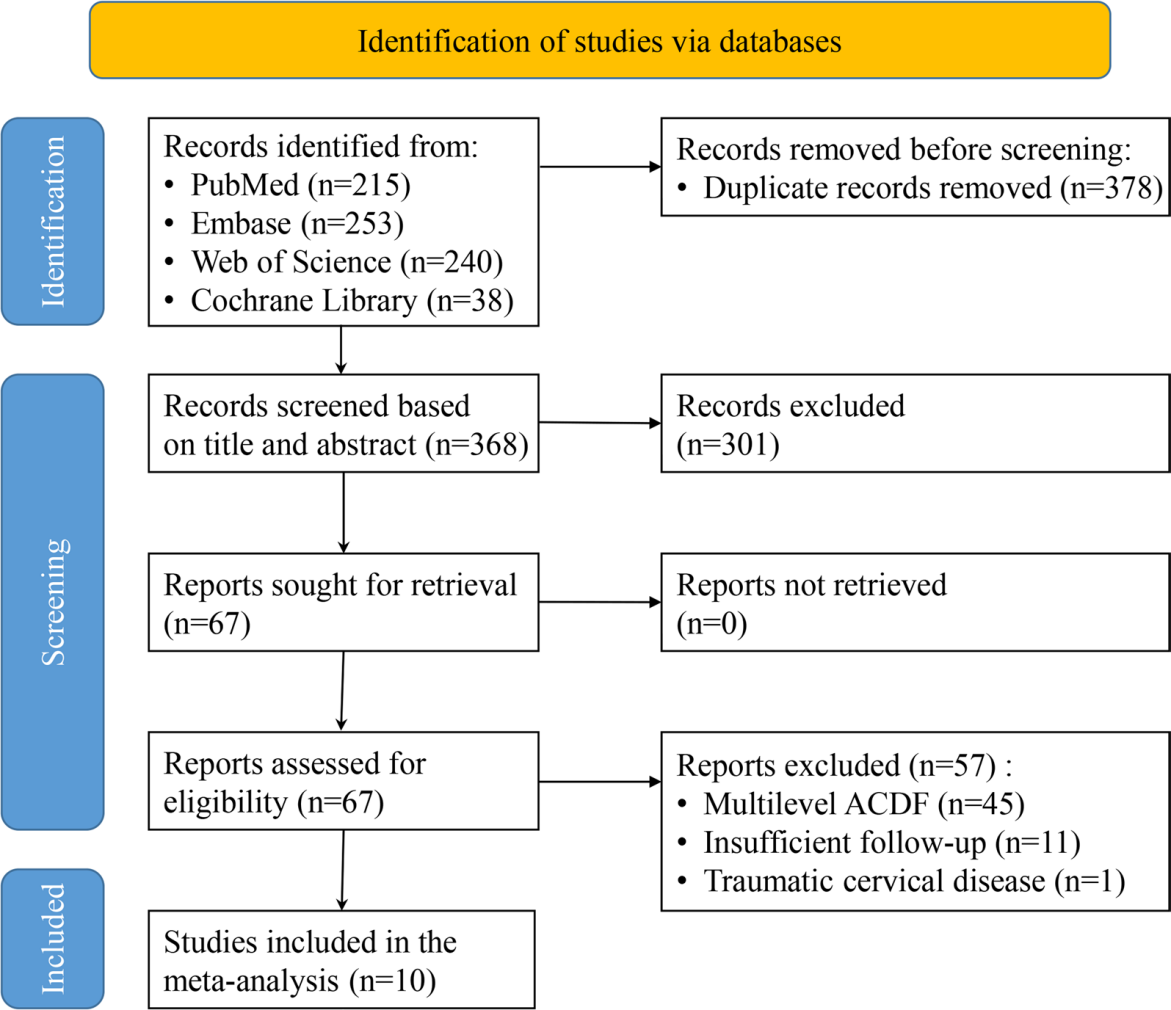
Flow chart of literature search

Initially, 746 relevant articles were selected through a comprehensive review of the electronic databases. Afterwards 378 duplicate records were removed. Subsequently, after scanning the titles and abstracts, 301 articles that failed to match the inclusion conditions were removed. Having read the full texts of the remaining 67 publications, ten of them were included for our study at last (Fig. 1) [21–30]. Among them, there were two randomised controlled trials and eight observational studies, which included a total of 979 patients who underwent monosegmental ACDF during 2009–2020 (Table 1).

**Table 1:** Study characteristics

Author	Publication year	Country	Study type	Sample size		Age (years)		Follow-up (months)	
				SSC	CPC	SSC	CPC	SSC	CPC
Lynch	2022	USA	OS	90	102	45.6 ± 8.5	48.1 ± 11.5	24	24
He	2021	China	OS	42	45	62.59 ± 8.21	61.15 ± 7.52	26.6 ± 3.3	27.1 ± 3.5
Noh	2021	Korea	OS	38	42	51.9 ± 10.21	52.6 ± 8.61	37.6 ± 15.91	37.1 ± 15.7
Zhang	2021	China	OS	74	116	50.14 ± 6.05	50.29 ± 9.06	34.07 ± 3.20	36.50 ± 6.28
Li	2020	China	OS	61	55	58.8 ± 4.6	58.5 ± 4.9	24	24
Lan	2018	China	OS	35	33	54.05 ± 10.11	52.09 ± 10.46	23.68 ± 1.93	24.39 ± 2.00
Noh	2018	Korea	OS	36	71	55.64 ± 10.31	55.06 ± 11.13	32.7 ± 17.5	32.7 ± 17.5
Li	2015	China	RCT	23	23	48.2 ± 7.9	49.2 ± 6.3	24	24
Nemoto	2015	Japan	RCT	24	22	40.9 ± 7.2	41.6 ± 7.0	24	24
Wang	2014	China	OS	22	25	50.86 ± 8.79	53.68 ± 8.96	33.59 ± 5.52	33.16 ± 5.97

*SSC*—self-locking stand-alone cage; *OS*—observational study; *RCT*—randomised controlled trial; *CPC*—cage-plate construct

### Quality assessment

**Fig. 2:**
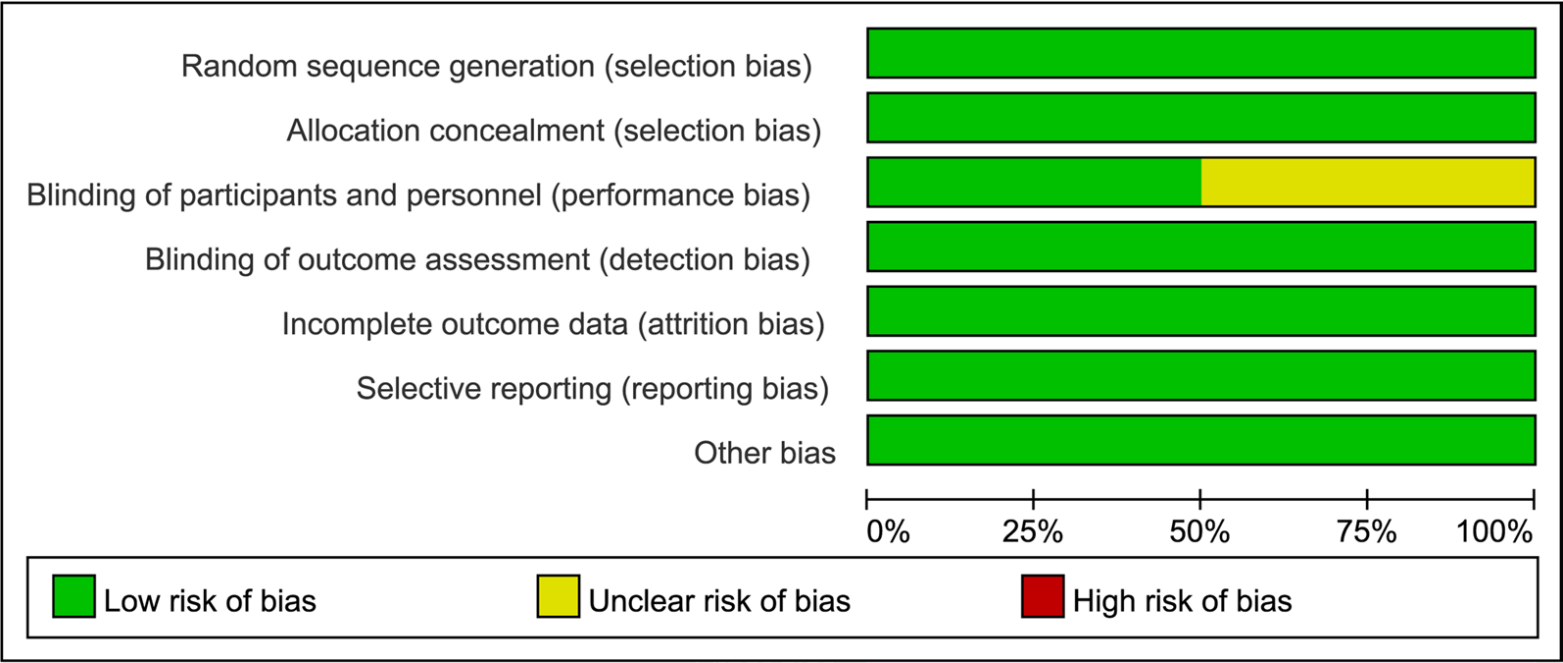
Cochrane risk of bias assessment for randomised controlled trials

According to the Cochrane Collaboration tools for assessing the risk of bias in two randomised controlled trials, the results are summarised in Figs. 2. One study did not adequately blind patients and doctors and therefore we considered performance bias as unclear.

In addition, we used the NOS scores to assess quality of observational studies. Detailed results of the quality assessment are shown in Table 2. The NOS results showed all articles scored at least 7 points and were considered to be of high quality.

**Table 2:** Newcastle–Ottawa scale of included observational studies

Study	Selection	Comparability	Outcome	Total score
Lynch [21]	4	1	3	8
He [24]	4	1	3	8
Noh [23]	3	1	3	7
Zhang [22]	4	1	3	8
Li [25]	3	1	3	7
Lan [27]	3	1	3	7
Noh [26]	4	1	3	8
Wang [30]	3	1	3	7

### Results of meta-analyses

#### Operation time

**Fig. 3:**
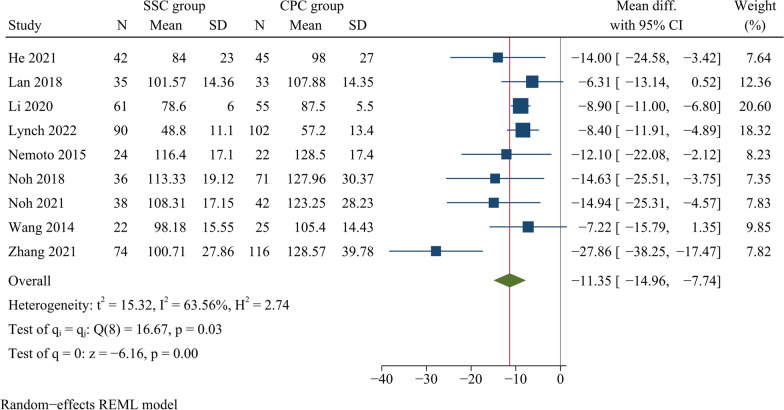
Forest plot of operation time

Nine studies compared operation time, including a total of 933 cases. The operation time was significantly shorter in the SSC group than that in the CPC group [WMD =  −11.35, 95% CI (−14.96, −7.74), *p* < 0.001, *I*^2^ = 63.56%] (Table 3) (Fig. 3).

**Table 3:** Results of meta-analyses

Outcomes	Study size	Effect size	95% CI		*p*-Value	Heterogeneity	Effect model	Egger’s test
		WMD/OR	Lower limit	Upper limit		I^2^ (%)		*p*-Value
Operation time	9	−11.35	−14.96	−7.74	< 0.001	63.56	Random	0.08
Intraoperative blood loss	9	−8.00	−11.61	−4.39	< 0.001	71.12	Random	0.10
Length of hospital stay	3	−1.53	−2.15	−0.91	< 0.001	34.71	Fixed	0.34
JOA score	4	−0.03	−0.30	0.24	0.82	0.00	Fixed	0.26
NDI score	4	0.32	−0.67	1.31	0.53	54.38	Random	0.22
Cervical Cobb angle								
One month postoperatively	4	−0.21	−1.04	0.62	0.61	0.00	Fixed	0.89
Final follow-up	8	−1.51	−2.16	−0.85	< 0.001	0.00	Fixed	0.08
Fusion	8	0.65	0.30	1.42	0.28	0.00	Fixed	0.72
Cage subsidence	5	0.84	0.48	1.49	0.56	0.00	Fixed	0.95
Adjacent segment degeneration	4	0.33	0.19	0.57	< 0.001	46.70	Fixed	0.25
Dysphagia								
One month postoperatively	7	0.30	0.18	0.50	< 0.001	0.00	Fixed	0.47
Final follow-up	4	0.30	0.06	1.42	0.13	0.00	Fixed	0.61

*WMD* weighted mean difference; *NDI* neck disability index; *OR* odds ratio; *JOA* Japanese orthopaedic association; *CI* confidence interval

#### Intraoperative blood Loss

**Fig. 4:**
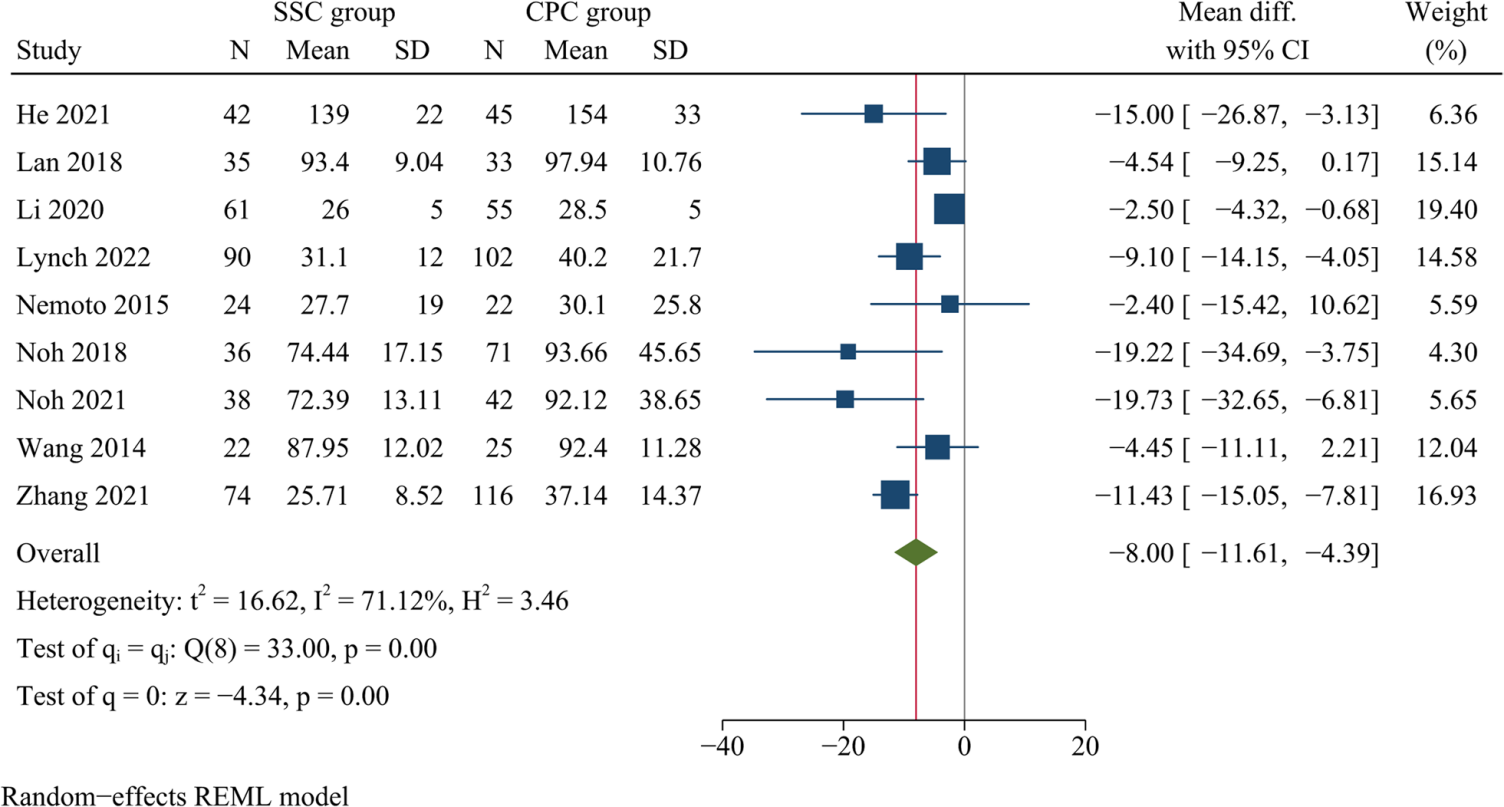
Forest plot of intraoperative blood loss

Nine publications, including 422 patients in the SSC group and 511 patients in the CPC group, reported intraoperative bleeding. Compared to the CPC group, the SSC group had less intraoperative blood loss and the difference was statistically significant [WMD = −8.00, 95% CI (−11.61, −4.39), *p* < 0.001, *I*^2^ = 71.12%] (Fig. 4).

#### Length of hospital stay

**Fig. 5:**
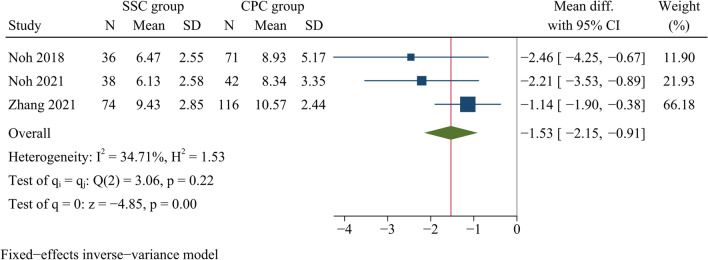
Forest plot of length of hospital stay

Three studies involving 377 patients compared the length of hospitalisation. Eventually, the result showed CPC was significantly related to a longer hospital stay in comparison with SSC [WMD =  −1.53, 95% CI (−2.15, −0.91), *p* < 0.001, *I*^2^ = 34.71%] (Fig. 5).

##### JOA scores

**Fig. 6:**
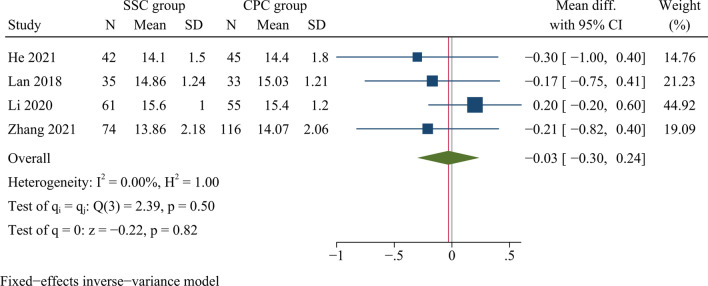
Forest plot of JOA scores at final follow-up

Four studies, involving a total of 461 cases, analysed JOA scores at final follow-up. Finally, it was concluded from the result that the JOA scores were not significantly different between SSC and CPC at final follow-up [WMD =  −0.03, 95% CI (−0.30, 0.24), *p* = 0.82, *I*^2^ = 0.00%] (Fig. 6).

##### NDI scores

**Fig. 7:**
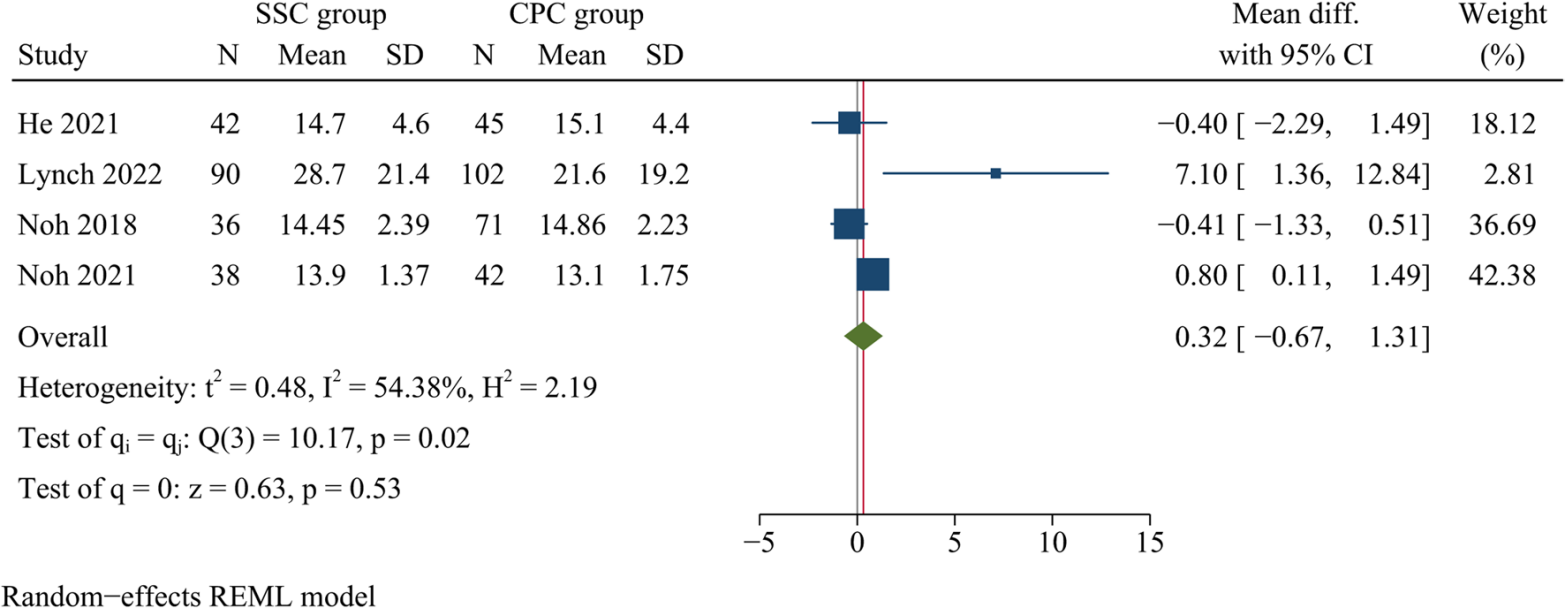
Forest plot of NDI scores at the final follow-up

NDI scores at the last follow-up were reported in four publications, including a total of 466 patients (206 in the SSC group and 260 in the CPC group). There was no significant difference regarding NDI scores at final follow-up between both groups [WMD = 0.32, 95% CI (−0.67, 1.31), *p* = 0.53, *I*^2^ = 54.38%] (Fig. 7).

#### Cervical curvature

**Fig. 8:**
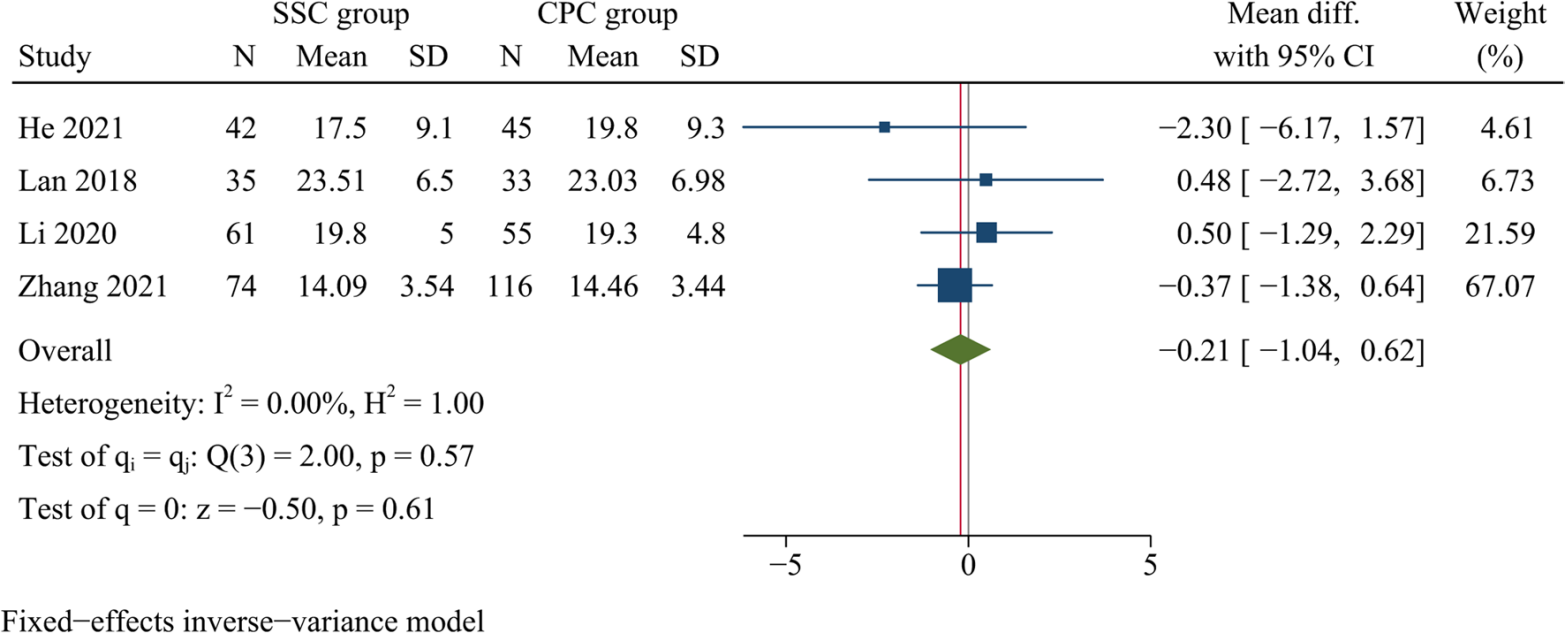
Forest plot of cervical Cobb angle at one month postoperatively

Four articles involving a total of 461 patients reported the cervical Cobb angle at one month postoperatively. Between the SSC and CPC groups, there was no significant difference regarding cervical Cobb angles at one month postoperatively [WMD =  −0.21, 95% CI (−1.05, 0.62), *p* = 0.61, *I*^2^ = 0.00%] (Fig. 8).

**Fig. 9:**
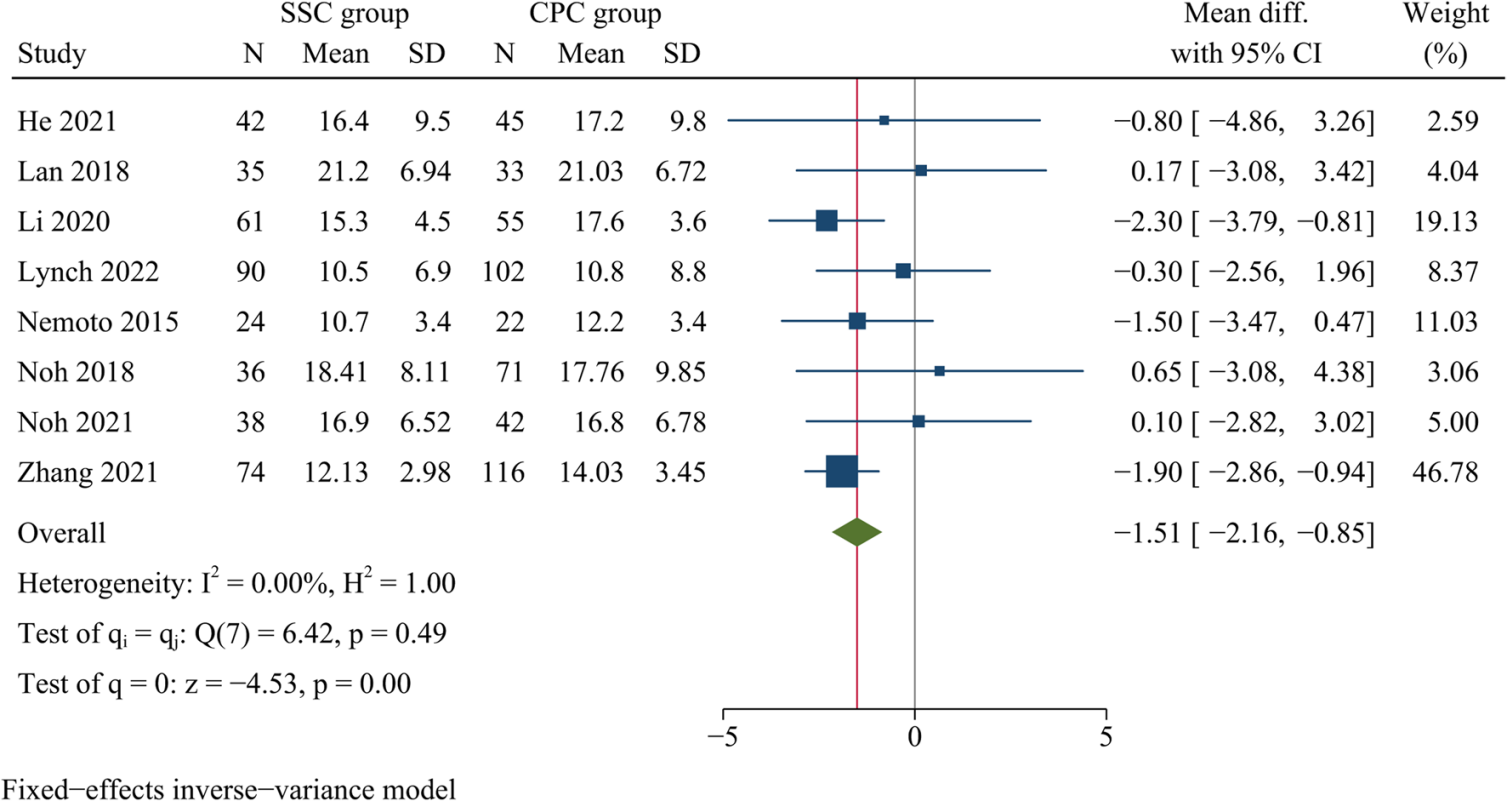
Forest plot of cervical curvature at final follow-up

Eight articles with a total of 886 patients (SSC group: 400; CPC group: 486) reported the cervical Cobb angle at the last follow-up. The cervical Cobb angle at last follow-up was significantly smaller in the SSC group than in the CPC groups [WMD =  −1.51, 95% CI (−2.16, −0.86), *p* < 0.001, *I*^2^ = 0.00%] (Fig. 9).

#### Intervertebral fusion rate

**Fig. 10:**
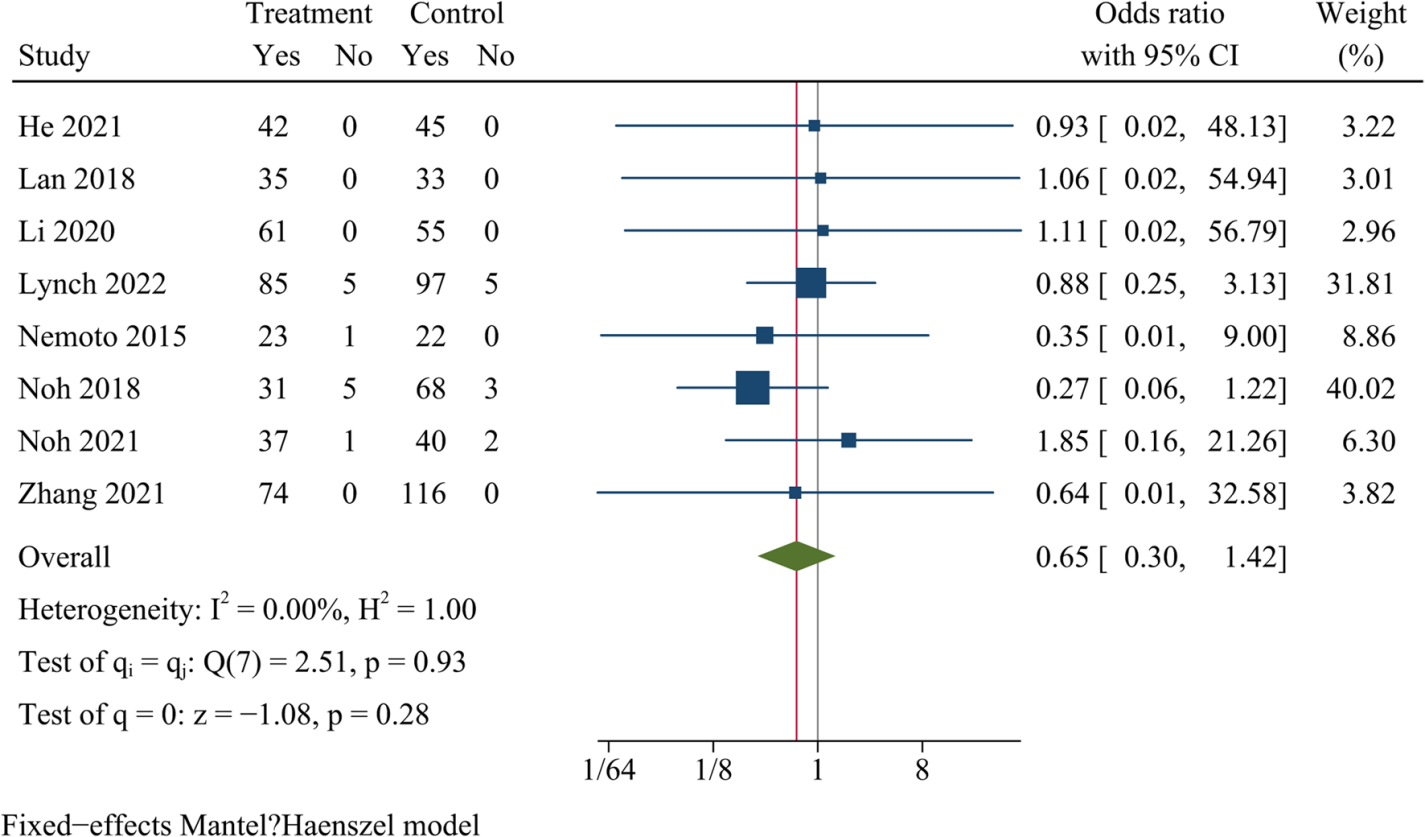
Forest plot of fusion rate

The fusion rate at the last follow-up was reported in eight studies in which a total of 886 cases were included. There was no significant difference in fusion rate between the SSC and CPC groups at the final follow-up [OR = 0.66, 95% CI (0.30, 1.42), *p* = 0.28, *I*^2^ = 0.00%] (Fig. 10).

#### Cage subsidence

**Fig. 11:**
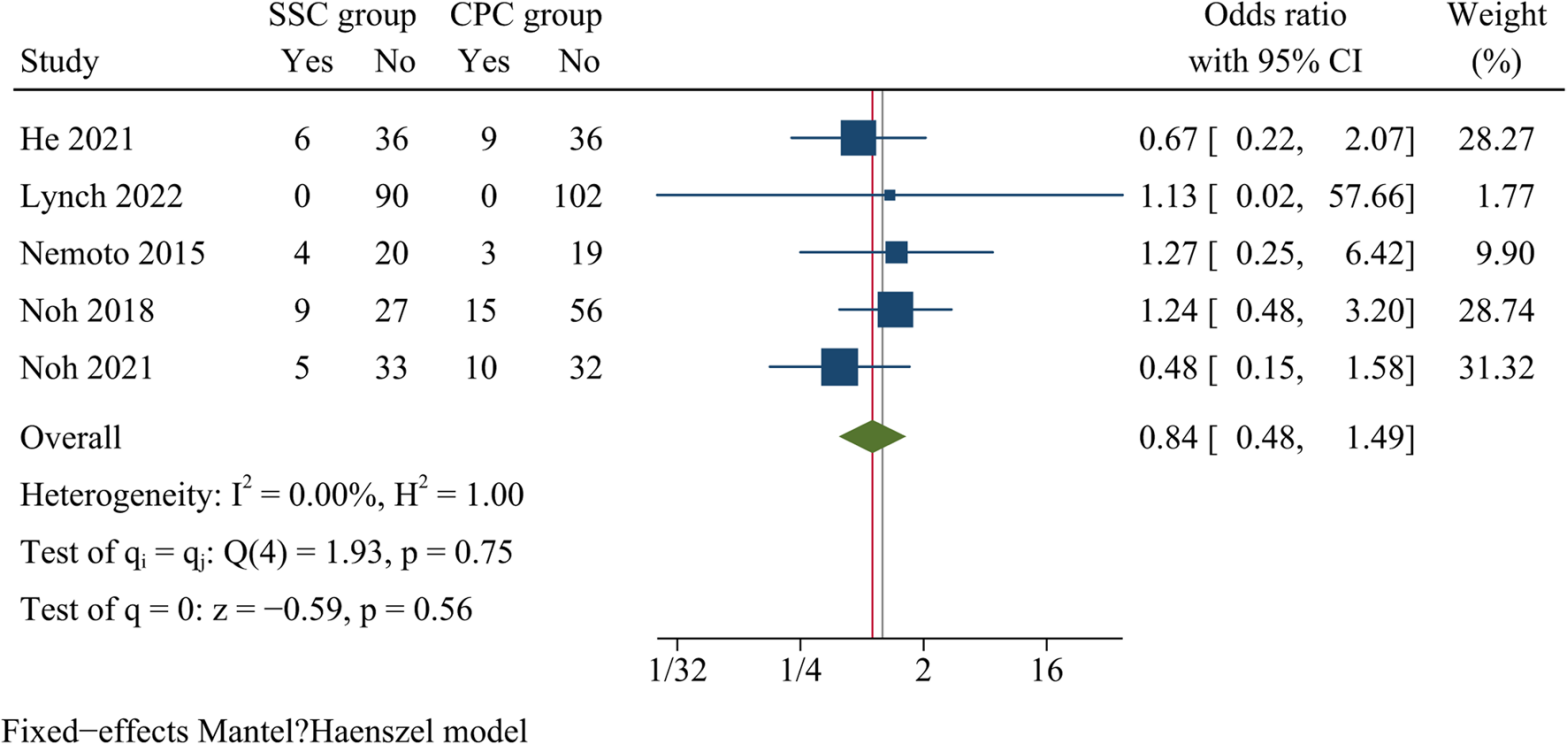
Forest plot of cage subsidence rates

Overall, 512 patients were analysed among five papers that reported data on the incidence of cage subsidence. The results showed no statistical difference with regard to cage subsidence rates between both groups [OR = 0.84, 95% CI (0.48, 1.49), *p* = 0.56, *I*^2^ = 0.00%] (Fig. 11).

#### Adjacent segment degeneration

**Fig. 12:**
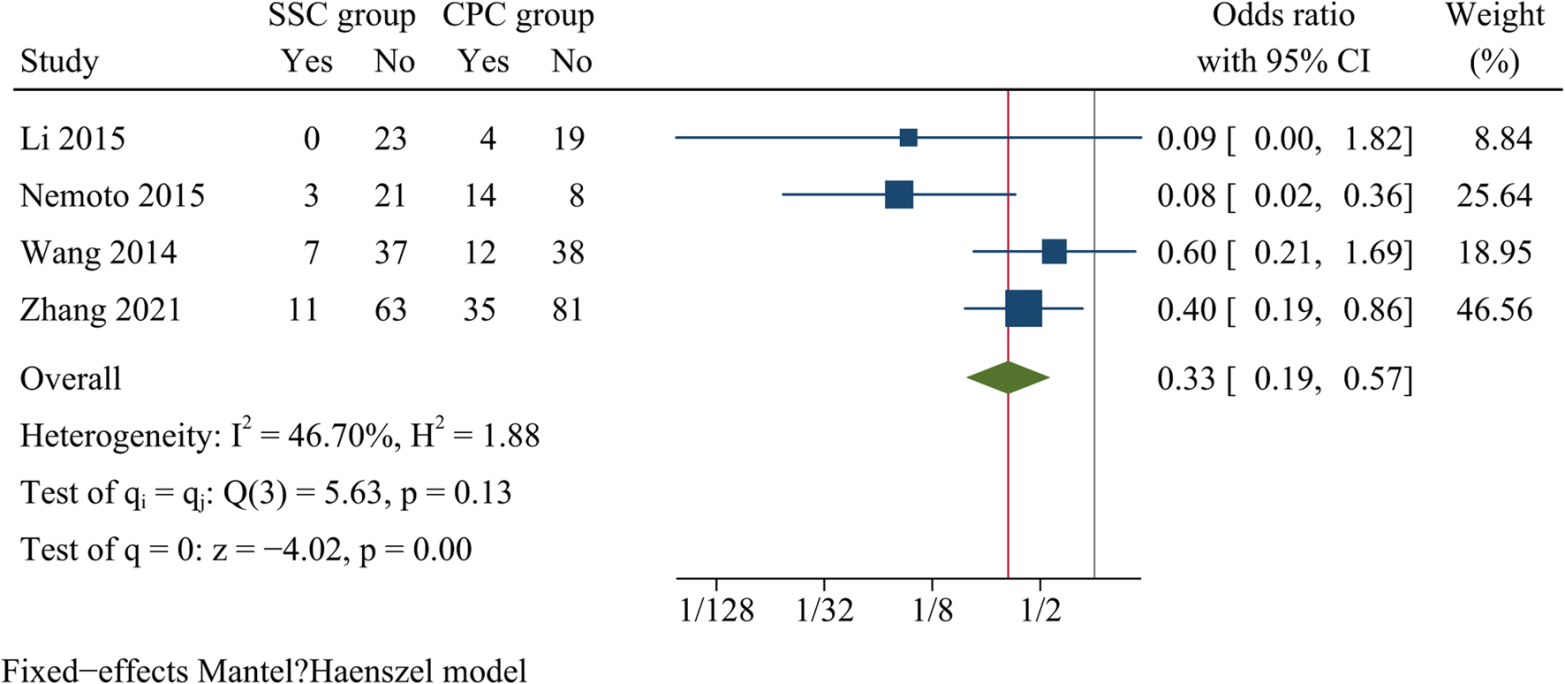
Forest plot of adjacent segment degeneration rate

ASD rate at final follow-up was reported in four publications. Analysis results showed that there were significant differences in ASD rate between the SSC and CPC groups, and the incidence of ASD was higher in the CPC group than in the SSC group [OR = 0.33, 95% CI (0.19, 0.57), *p* < 0.001, *I*^2^ = 46.70%] (Fig. 12).

#### Dysphagia

**Fig. 13:**
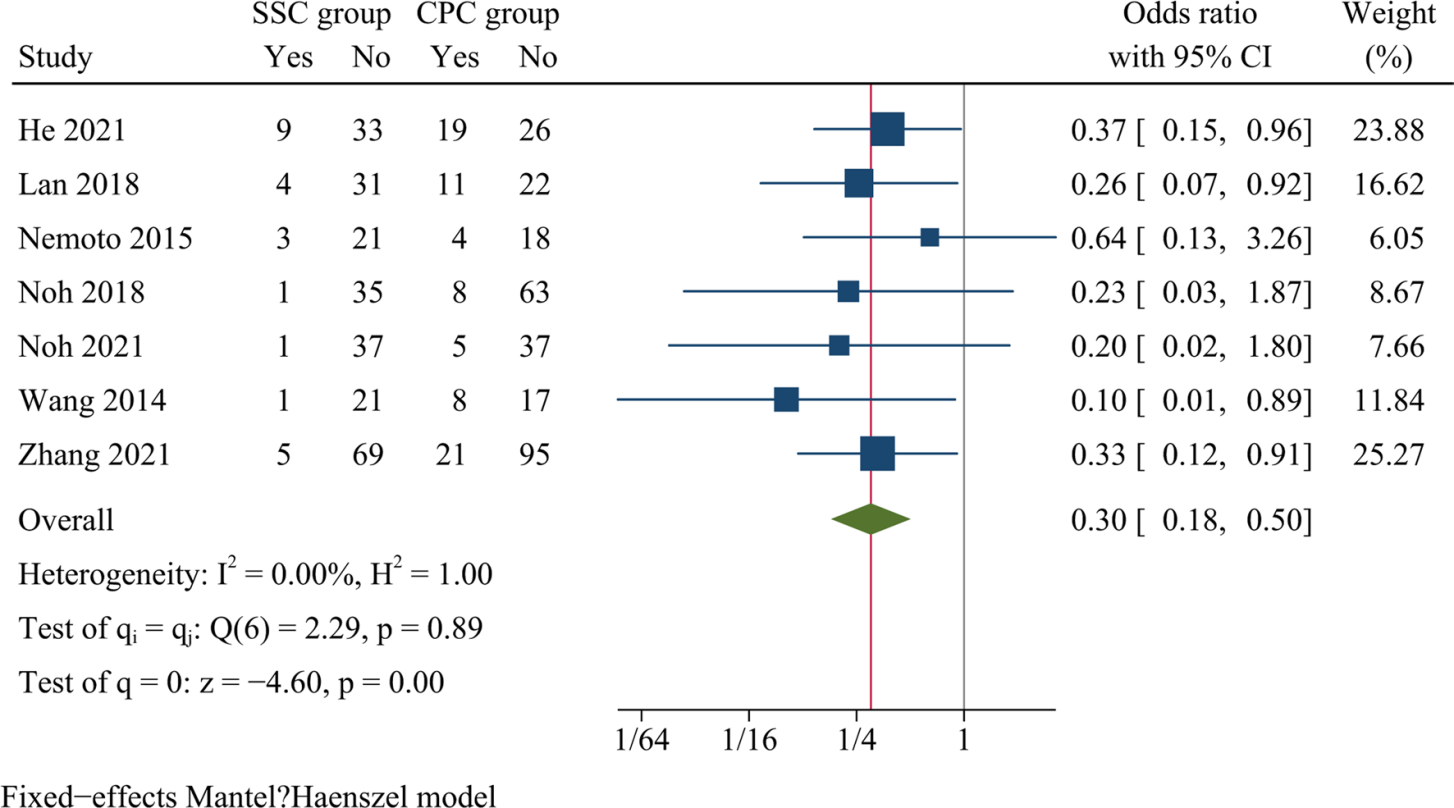
Forest plot of dysphagia rate at one month postoperatively

**Fig. 14:**
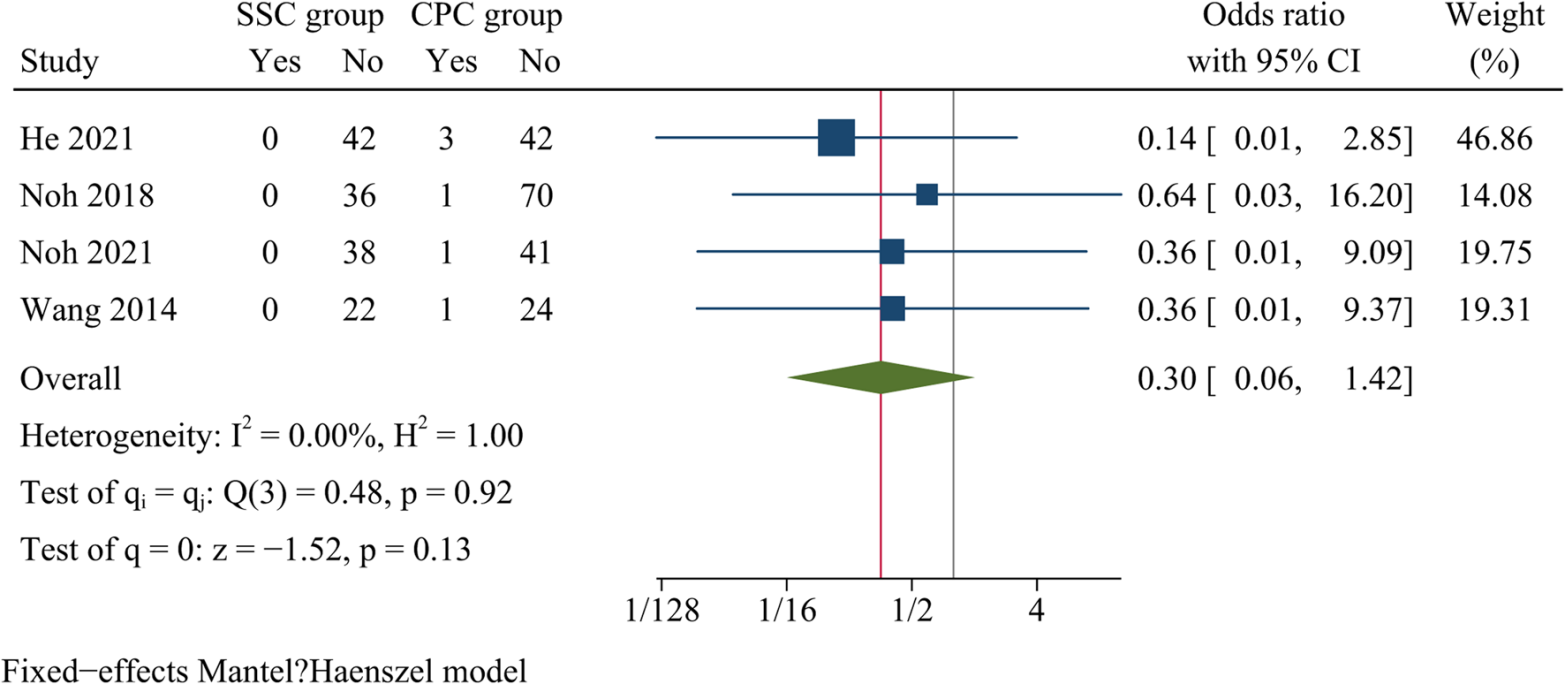
Forest plot of dysphagia rate at the final follow-up

Postoperative dysphagia rates at one month after surgery and at the last follow-up were reported in seven and four studies, respectively. The findings showed there was statistically significant difference concerning dysphagia rates at one month after surgery between SSC and CPC groups [OR = 0.30, 95% CI (0.18, 0.50), *p* < 0.001, *I*^2^ = 0.00%] (Fig. 13). Whereas there was no significant difference concerning dysphagia rates between both groups at final follow-up [OR = 0.30, 95% CI (0.06, 1.42), *p* = 0.13, *I*^2^ = 0.00%] (Fig. 14).

#### Sensitivity analysis

To perform sensitivity analyses, individual studies were eliminated in turn and the remaining studies were pooled. Finally, effect sizes of the outcome measures had no statistically significant change after removal of the individual studies. Hence, the pooled results of this study were robust (Additional file 2).

#### Meta-regression for the potential sources of heterogeneity

There was significant heterogeneity among studies with regard to operation time, intraoperative blood loss and NDI scores at the final follow-up. Therefore, through the univariate meta-regression analysis, we further explored the sources of heterogeneity. Nevertheless, the findings indicated publication year, study region and study type were not contributing to heterogeneity (*p* > 0.05) (Additional file 3). Consequently, no subgroup analysis was carried out.

#### Publication bias assessment

Egger's test was used to detect publication bias. The results showed no significant publication bias among all outcomes (*p* > 0.05) (Additional file 4).

## Discussion

### Background and main findings

ACDF is currently the standard operation for the treatment of DCS, which can fully decompress during the operation, restore cervical curvature, and improve the neurological function of patients. At present, there are two main types of cervical fixation devices: CPC and SSC, with their corresponding pros and cons. With regard to clinical effectiveness, the choice between the two devices remains highly controversial [13, 15]. Most of the current comparative studies between SSC and CPC have investigated short-term and medium-term outcomes. However, few studies reported the long-term outcomes of both devices in ACDF. The lack of adequate long-term follow-up means that the evidence is often not very convincing or robust. At the same time, different surgical segments may bring biomechanical bias to the results of the studies and become one of their limitations. Therefore, this study included studies with a follow-up of at least two years to objectively compare the long-term efficacy and safety between SSC and CPC in the treatment of monosegmental ACDF with the aim of providing an evidence-based medical basis for the selection of appropriate implants in monosegmental ACDF. Our findings showed that operative time, intraoperative blood loss, length of hospital stay, cervical curvature at last follow-up, incidence of ASD and dysphagia at one month postoperatively were significantly lower in the SSC group than in the CPC group. There were no significant differences between both groups with respect to JOA scores at last follow-up, NDI scores at last follow-up, cervical curvature at one month postoperatively, intervertebral fusion rate, incidence of cage subsidence or dysphagia at last follow-up. Our study was performed in accordance with the Preferred Reporting Items for Systematic Reviews and Meta-Analysis (PRISMA) Statement (Additional file 5).

### Interpretation of the results

#### Clinical outcomes

Our results showed that, compared with CPC, SSC significantly reduced operation time, intraoperative bleeding, and length of hospitalisation. However, there was significant heterogeneity regarding operation time and intraoperative blood loss. Therefore, we used meta-regression analysis to explore possible factors contributing to heterogeneity, such as publication year, study region and study design. However, the results showed that the factors mentioned above were not responsible for the heterogeneity. Consequently, we did not perform a subgroup analysis. Clinically, surgical duration and intraoperative bleeding are usually related to the experience and surgical habits of the surgeon, which can lead to heterogeneity. Zhang et al. found that SSC reduced operative time significantly compared with CPC, but no significant difference was found in intraoperative bleeding between both groups [4]. Li et al. reported that SSC had a shorter surgical duration and less intraoperative blood loss than CPC [31]. Sommaruga et al. found that patients with CPC had a significantly longer surgical and hospitalisation than those with SSC [11]. A meta-analysis based on randomised controlled trials showed that surgical duration and intraoperative bleeding were significantly reduced with SSC compared to CPC for monosegmental ACDF [20]. Fixation with a titanium plate requires extensive exposure of the surgical field for plate placement. In contrast, SSC requires only a small, minimally invasive incision of 3–5 cm to expose the pathological intervertebral disc without over-exposing the adjacent vertebral body. In addition, the relatively uniform angle of screw insertion simplifies self-locking screw fixation. These above advantages technically reduce the operative time and intraoperative blood loss and help to facilitate postoperative recovery and shorten the duration of hospitalisation.

The main criteria for evaluating the effectiveness of surgery are the adequacy of nerve decompression and recovery of nerve function after surgery. The main objective of surgery is to decompress the compressed spinal cord and nerve roots sufficiently to create favourable conditions for nerve recovery. Direct decompression of the nerve by ACDF has proven to be effective in the treatment of DCS. Several studies reported no significant differences between the two interbody fusion systems for the treatment of DCS as to JOA and NDI scores at final follow-up, which indicated that both devices had similar satisfactory efficacy [13, 15, 32]. Overall, we found no significant differences between SSC and CPC concerning JOA scores and NDI scores at the final follow-up. This result indicated that in the long term, SSC could achieve the same neurological recovery as CPC.

#### Radiological outcomes

In our study, the fusion rate at the last follow-up was similar between both groups. It was reported that there was no significant difference in bone fusion rate at 12 months after surgery between the SSC and CPC groups in monosegmental ACDF [4]. Zhu et al. found that fusion rates in both groups were similar at 3-year follow-up after multilevel ACDF [33]. The above findings were consistent with those of Guo et al.[19]. SSC can be placed directly into the intervertebral space and be anchored to the adjacent vertebral body with screws through the endplate, providing biomechanical stability similar to titanium plates and improving fusion rates. Furthermore, effective interbody bone fusion can prevent the fusion devices from loosening or sinking, thereby helping to maintain cervical sagittal alignment and reduce postoperative loss of cervical curvature. Hence, the result demonstrated that the SSC system could achieve a satisfactory fusion rate in the long term, similar to that achieved by the CPC system.

Regarding the cervical Cobb angle, our results showed no significant difference between the SSC and CPC groups at one month postoperatively. However, the cervical Cobb angle at the final follow-up was significantly smaller in the SSC group than in the CPC group. A meta-analysis by Kahaer et al. found no significant difference in cervical curvature at final follow-up between SSC and CPC. The disagreement with our results might be explained by the fact that some of their included studies had a follow-up period of less than two years [18]. Liu et al. found that cervical curvature was greater in the CPC group than in the SSC group at both postoperative and final follow-up in their meta-analysis, which, nevertheless, included both single- and multi-segment ADCF [17]. Further subgroup analyses showed that in monosegmental ACDF, postoperative cervical curvature was not significantly different between both groups, whereas CPC had greater cervical curvature at final follow-up than SSC. This finding was similar to our results. Furthermore, according to different time points of last follow-up, the subgroup analysis showed that the cervical curvature was significantly less in the SSC group than in the CPC group at the last follow-up of more than three years, which indicated that as follow-up duration became longer, the cervical curvature in the SSC group would decrease significantly, compared to that in the CPC group. In addition, Guo et al. carried out a retrospective cohort study concerning 3-level ACDF and found that there were no significant differences between both fusion devices in terms of JOA scores, NDI scores and fusion rates at the last follow-up, but the traditional plate-cage system had a better cervical Cobb angle than the SSC system at the 2-year follow-up after surgery [13]. Although the exact pathophysiological mechanism of postoperative cervical curvature loss remained unknown, several biomechanical studies found that anterior plate fixation could provide better cervical stability than SSC [34, 35]. Our findings confirmed that in monosegmental ACDF, the cervical curvature at long-term follow-up was lower in the SSC group than in the CPC group. Despite this, we consider that symptom relief is more directly related to the degree of decompression and whether radiological changes affect clinical symptoms should be confirmed in trials with longer follow-up.

#### Complications

Dysphagia is the most common complication after ACDF surgery, which is usually thought to be related to the use of titanium plates and oesophageal traction during the operation. Yet the exact pathophysiological mechanism of dysphagia remains unknown. Lee et al. believed the thickness of titanium plates was an important risk factor for dysphagia after surgery and suggested that smaller, smoother titanium plates could significantly reduce the incidence of this complication [36]. Studies have shown that the usage of SSC in ACDF can considerably decrease the occurrence of postoperative dysphagia [4, 7]. We found the incidence of dysphagia was significantly lower in the SSC group than that in the CPC group at one month postoperatively, which might be because that the zero-profile design of SSC could avoid direct contact between the traditional titanium plate and the oesophagus, reducing postoperative irritation to the oesophagus from the embedded implants. Compared with conventional titanium plates, placement of SSC was easier and required less exposure, resulting in shorter operative durations, less bleeding and less intraoperative muscle traction damage. These advantages therefore reduced local tissue oedema, inflammatory response and oesophageal irritation [37, 38]. Besides, we found no significant difference in the incidence of dysphagia between the two groups at final follow-up, which might be related to postoperative regression of soft tissue oedema in the anterior cervical spine, patient adaptation and functional recovery. Similar results were reported by Liu et al. They concluded that patients in the CPC group had a prolonged recovery phase from dysphagia [17]. Overall, the application of SSC is more advantageous than CPC in preventing postoperative dysphagia in monosegmental ACDF.

Adjacent segment degeneration is considered to be another common complication of ACDF. Biomechanical studies showed that the use of titanium plates increased stress on the adjacent disc and accelerated degeneration of the adjacent segments [39, 40]. It was reported that a plate-to-disc distance of < 5 mm was a risk factor for ASD in ACDF surgery [41]. According to a study by Zhou et al., the risk of postoperative ASD was significantly higher in the CPC group than in the SSC group [8]. The result might be due to the fact that SSC was completely placed in the intervertebral space, distant to the contiguous levels, and micromotion of fused intervertebral space reduced the pressure on the adjacent segments, thus reducing the potential for post-fusion degeneration of the adjacent segments. Similar findings were observed in previous meta-analyses [17–19]. Likewise, our results showed that the incidence of ASD at final follow-up was significantly higher in the CPC group than in the SSC group, indicating that SSC was advantageous over CPC in lowering the long-term occurrence of ASD in monosegmental ACDF.

Apart from the complications mentioned above, SSC has an additional concern related to cage subsidence. Previous meta-analyses showed that SSC had a higher rate of subsidence than anterior plate fixation [16, 42]. In contrast, researches by Zhao et al. and Kahaer et al. found no differences in subsidence rates between the two devices [18, 20]. The reason for the different results in the above meta-analyses might be due to the inclusion of studies with different segments and follow-up periods. A retrospective cohort study demonstrated cage subsidence rates were significantly higher in the SSC group than in the CPC group in multi-segment anterior surgery, whereas in contrast the difference was not significant in single-segment operations [14]. Baesd on the results of our study, we concluded that there were no significant differences in cage subsidence rates at final follow-up between the SSC and CPC groups, probably due to the similar biomechanical properties and similar fusion rates of the two devices. Our results demonstrated that the use of SSC in monosegmental ACDF did not increase the risk of device subsidence compared with CPC, even beyond two years of follow-up, indicating the long-term safety of SSC in monosegmental ACDF.

## Strengths and limitations

Our meta-analysis has several advantages. First of all, we established strict criteria for study selection. This study focused only on the most common patients with single-level cervical spondylosis in the clinic, thus eliminating the bias caused by the number of segments in the overall analysis. Meanwhile, in order to more fully compare the long-term efficacy and safety between SSC and CPC, only studies with a follow-up of ≥ 2 years were included in the meta-analysis. Based on the strict inclusion and exclusion criteria of our study, potential confounding factors were eliminated as much as possible to improve the validity and reliability of the results in our study. In addition, Egger's test was carried out in this study, which proved that the possibility of publication bias was not significant. Therefore, our study has important clinical value and deserves careful interpretation, which can provide clinicians with more reliable evidence in treating monosegmental ACDF. Also, the results of this study may provide clues and guidance for future large sample, high quality randomised controlled trials in terms of study design.

This study also has some limitations. Firstly, the number of included studies was small, with only two RCTS. The included studies were primarily observational, which reduced our ability to account for the different clinical heterogeneity inherent in ACDF surgery and the selection bias between the SSC and CPC groups. Secondly, in terms of some outcome indicators, there was significant heterogeneity between the studies. However, meta-regression analysis failed to find the potential source of heterogeneity and we failed to conduct subgroup analysis. Thirdly, the types of SSC included in the study were inconsistent, which could lead to the bias of the results. Fourthly, all included studies were conducted in the English language. Therefore, there might be an underlying linguistic bias. Finally, due to the lack of medical expenditure in the papers included in this study, the advantages and disadvantages of the two devices could not be comprehensively compared. Therefore, in view of the shortcomings of the literature included in this study, the above findings need to be further verified by randomised controlled trials with large samples, multi-centre, long-term follow-up and high quality.

## Conclusions

Our analysis showed that compared with CPC, SSC achieved similar long-term effectiveness and safety in monosegmental ACDF in terms of JOA scores, NDI scores, fusion rate and cage subsidence rate at the end of follow-up. Notably, SSC had significant advantages over traditional ACDF in terms of reducing operative time, intraoperative blood loss, length of hospital stay, and the incidence of postoperative dysphagia and adjacent segment degeneration. Therefore, SSC appears to be preferable to CPC for patients who require monosegmental ACDF. However, SSC is inferior to CPC in maintaining cervical curvature at long-term follow-up. Given the limitations and potential other biases in our study, more large-scale, prospective, randomised controlled trials need to be undertaken to provide further evidence to validate our findings.

## Supplementary Information

**Additional file 1**: Search strategy.

**Additional file 2**: Sensitivity analysis.

**Additional file 3**: Meta-regression analysis for the potential sources of heterogeneity.

**Additional file 4**: Publication bias assessment.

**Additional file 5**: PRISMA checklist.

## Data Availability

Data supporting the findings of this study can be found in the article or its supplementary material. The protocol of this systematic review and meta-analysis is available in the Prospective Register of Systematic Reviews (PROSPERO) under number CRD 42022373028.

## Abbreviations

DCS: Degenerative cervical spondylosis
ACDF: Anterior cervical discectomy and fusion
CPC: Cage-plate construct
ASD: Adjacent segment degeneration
SSC: Self-locking stand-alone cage
OS: Observational studies
RCT: Randomised controlled trials
JOA: Japanese orthopaedic association
NDI: Neck disability index
NOS: Newcastle–Ottawa scale
OR: Odds ratios
CI: Confidence interval
WMD: Weighted mean difference
