# Supplementary material for: Self-locking stand-alone cage versus cage-plate fixation in monosegmental anterior cervical discectomy and fusion with a minimum 2-year follow-up: a systematic review and meta-analysis
Source: J Orthop Surg Res. 2023 Jun 2;18:403. doi: 10.1186/s13018-023-03885-4 (PMC10236847; doi:10.1186/s13018-023-03885-4)
Supplement: Supplementary file 1 — Additional file 1: Search strategy. [file 13018_2023_3885_MOESM1_ESM.pdf]

Search syntax of Pubmed, Web of Science and Cochrane Library:

((((((((((zero profile) OR (zero-profile)) OR (zero-p)) OR (no-profile)) OR (Stand-alone)) OR (anchored spacer)) OR (anchored cage)) OR (Self-locking)) OR (ROI-C)) OR (Prevail)) AND ((anterior cervical discectomy and fusion) OR (ACDF))) AND (plate)

Search syntax of Embase:

Embase

Embase session results (30 Jan 2023)

No.	Query	Results
#4	#1 AND #2 AND #3	253
#3	plate	186059
#2	anterior AND cervical AND discectomy AND fusion OR acdf	5677
#1	zero AND profile OR 'zero profile' OR 'zero p' OR 'no profile' OR 'stand alone' OR (anchored AND spacer) OR (anchored AND cage) OR 'self locking' OR 'roi c' OR prevail	27159
