# Supplementary material for: Self-locking stand-alone cage versus cage-plate fixation in monosegmental anterior cervical discectomy and fusion with a minimum 2-year follow-up: a systematic review and meta-analysis
Source: J Orthop Surg Res. 2023 Jun 2;18:403. doi: 10.1186/s13018-023-03885-4 (PMC10236847; doi:10.1186/s13018-023-03885-4)
Supplement: Supplementary file 2 — Additional file 2: Sensitivity analysis. [file 13018_2023_3885_MOESM2_ESM.pdf]

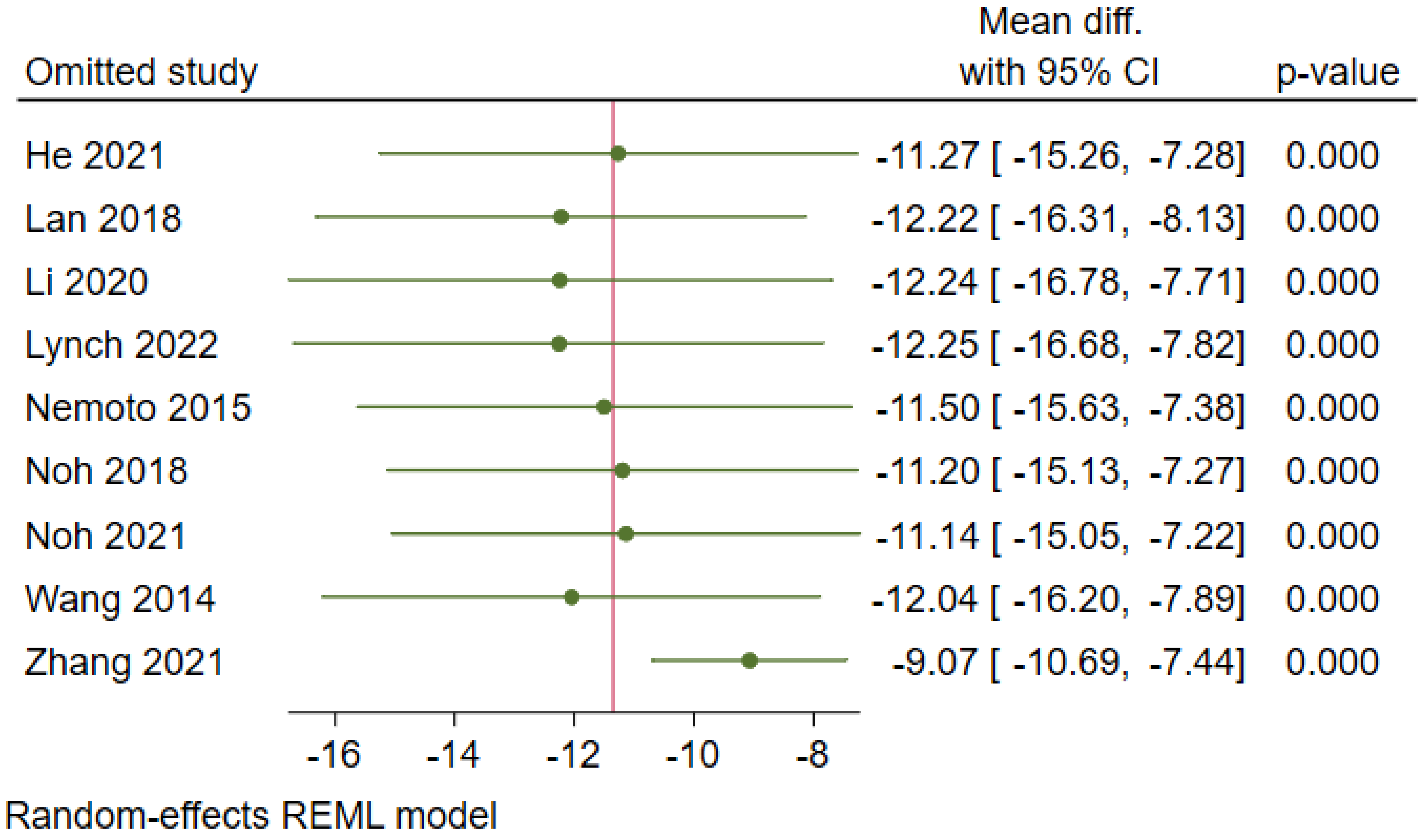

Sensitivity analysis for operation time

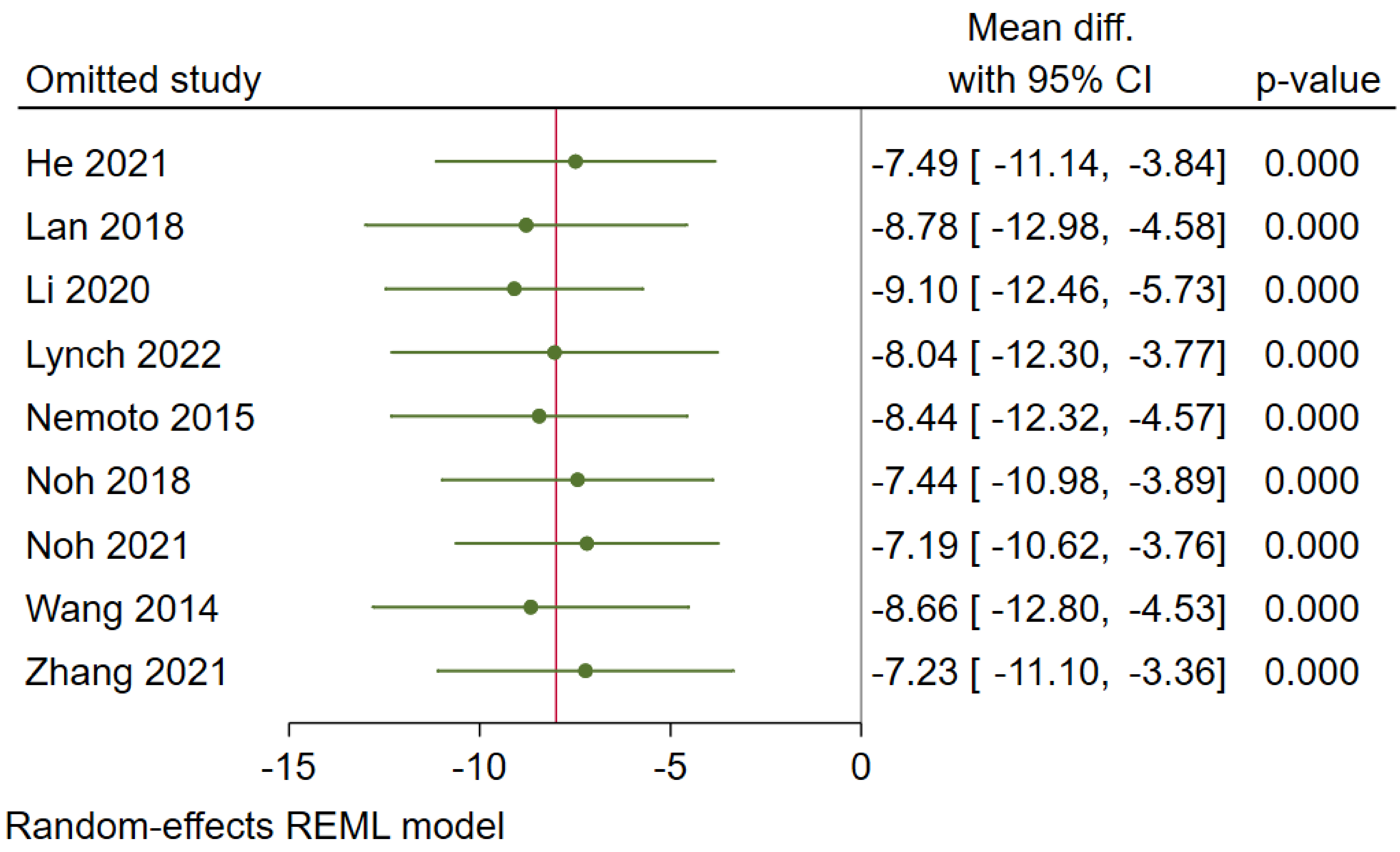

Sensitivity analysis for intraoperative blood loss

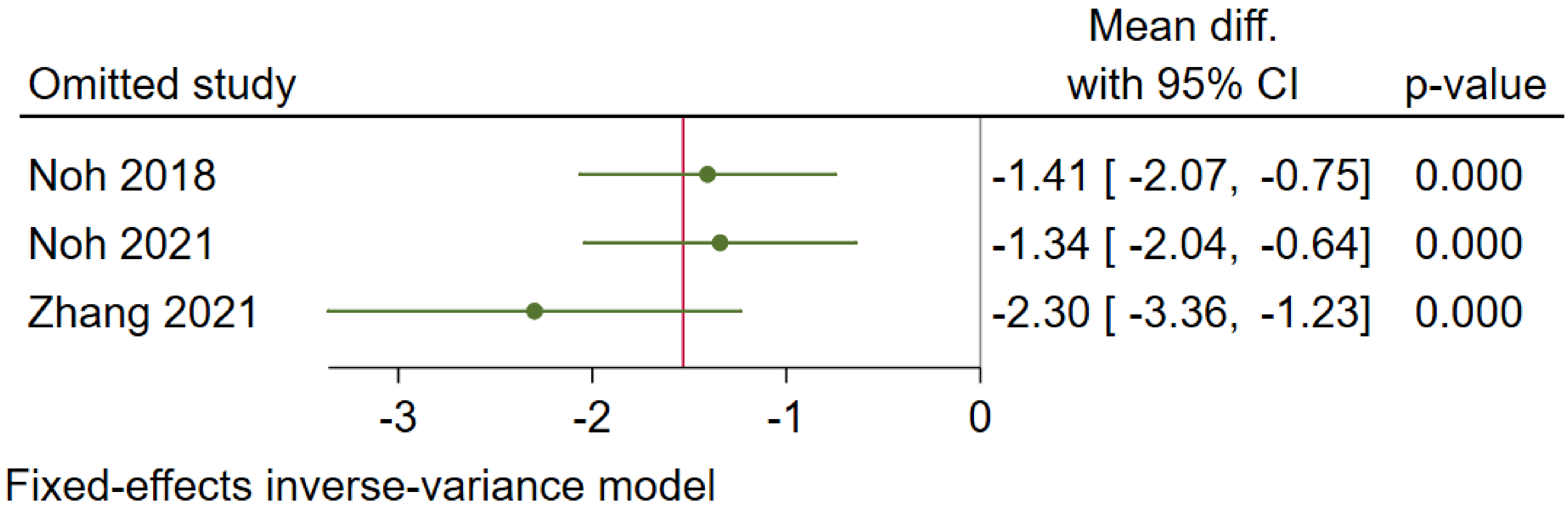

Sensitivity analysis for hospital stay

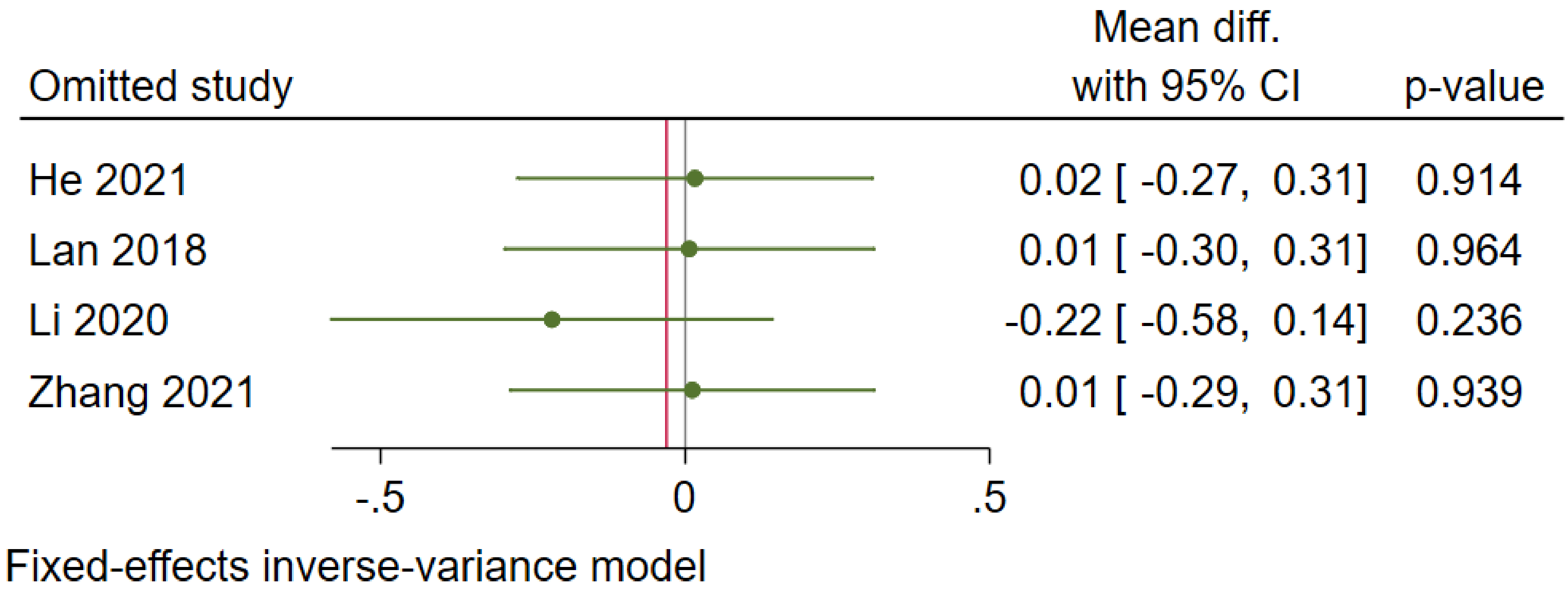

Sensitivity analysis for JOA scores

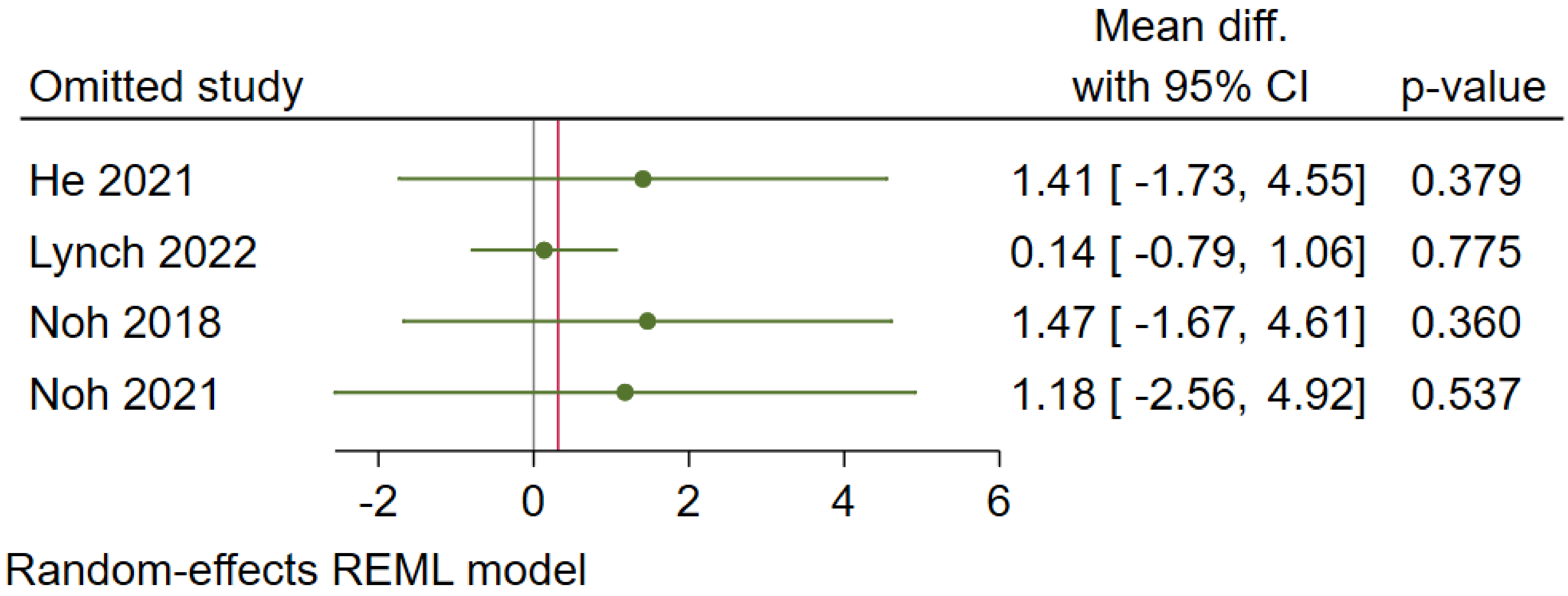

Sensitivity analysis for NDI scores

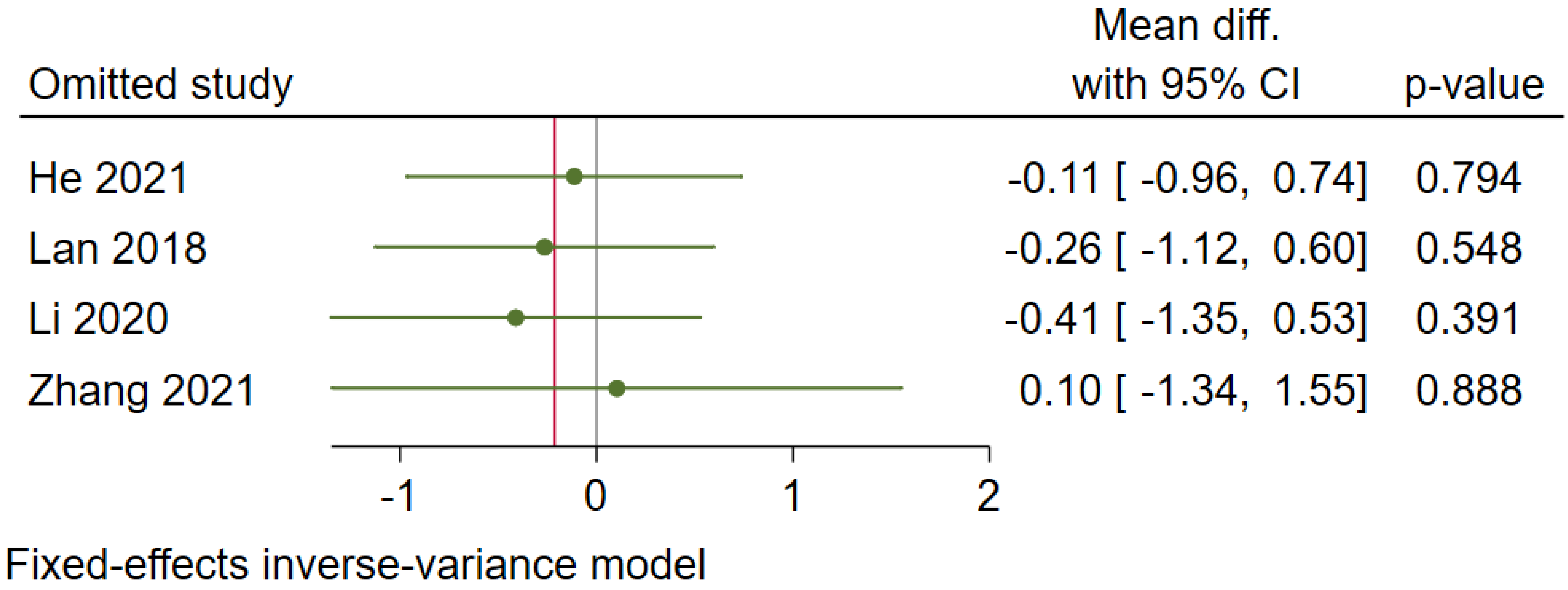

Sensitivity analysis for cervical Cobb angle at one month postoperatively

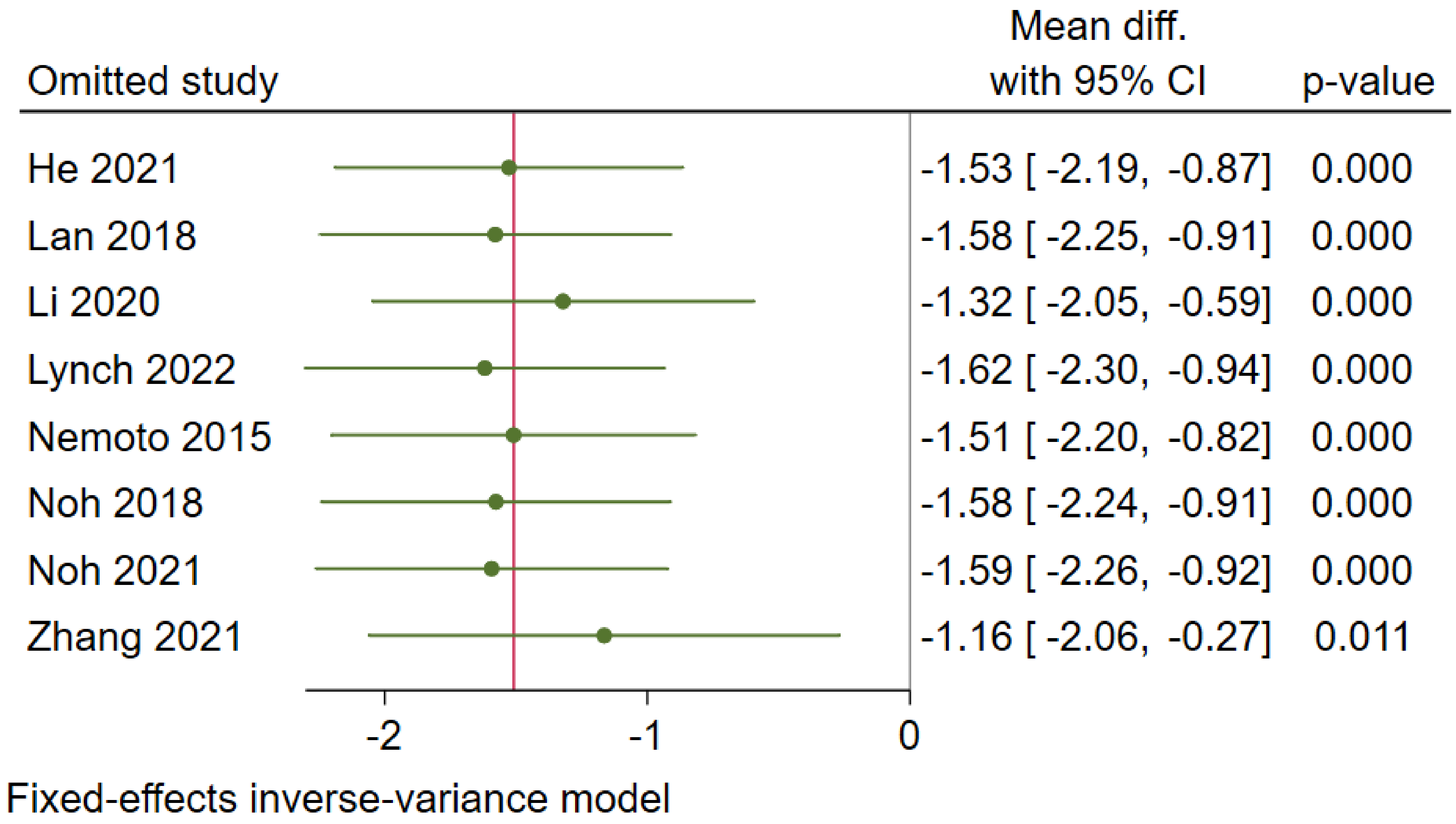

Sensitivity analysis for cervical Cobb angle at final follow-up

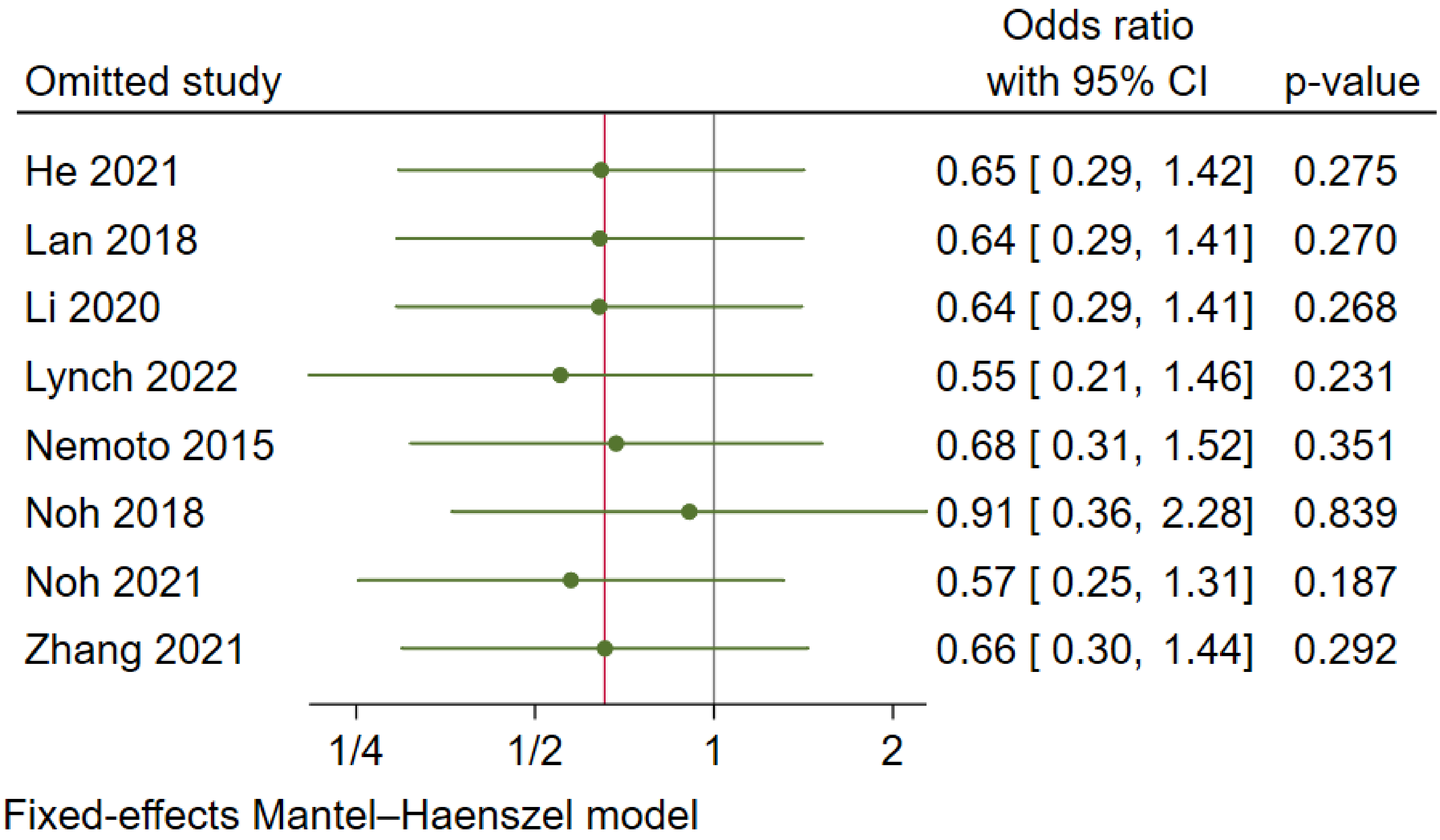

Sensitivity analysis for fusion rate

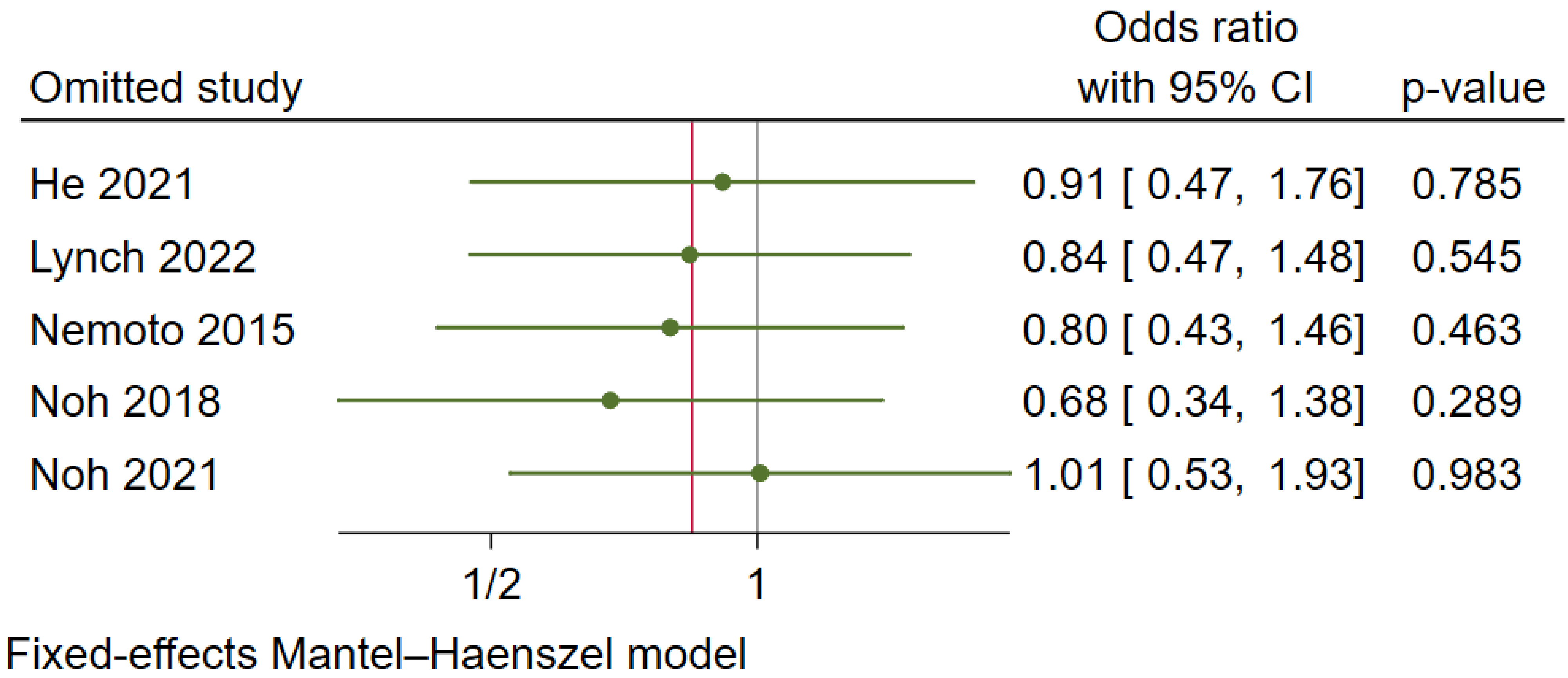

Sensitivity analysis for cage subsidence

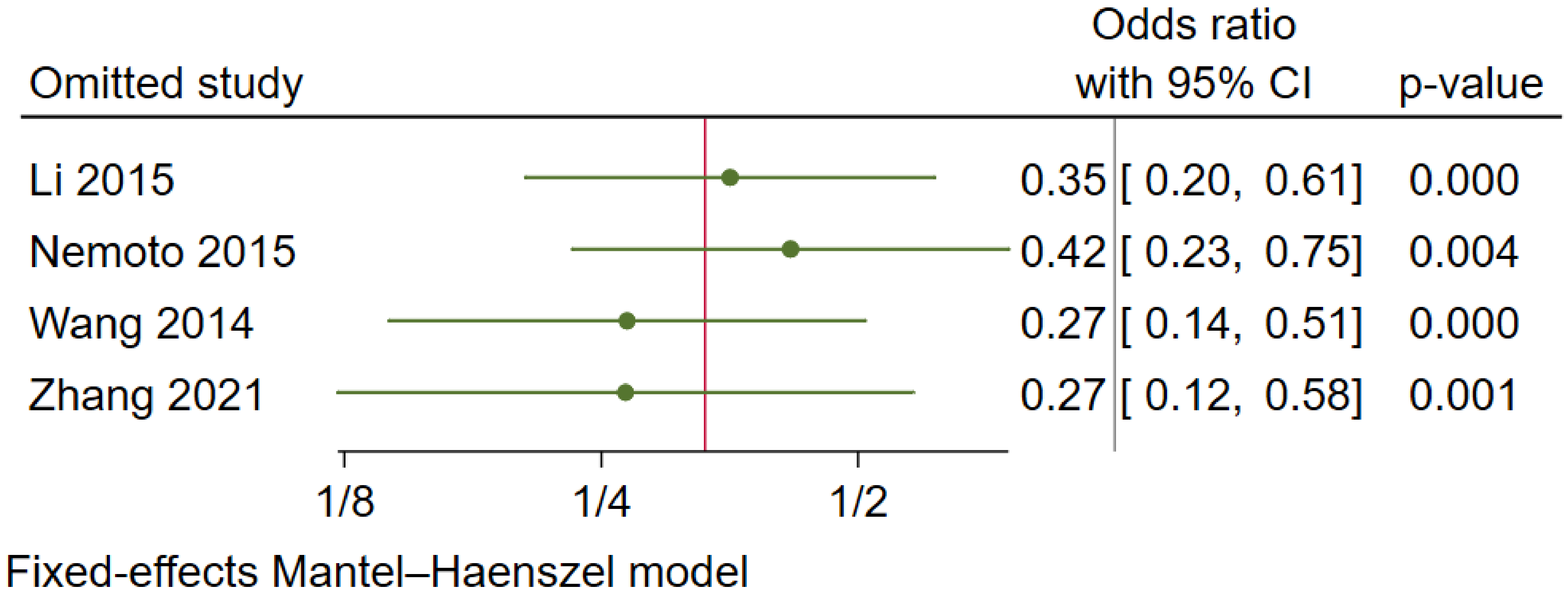

Sensitivity analysis for adjacent segment degeneration

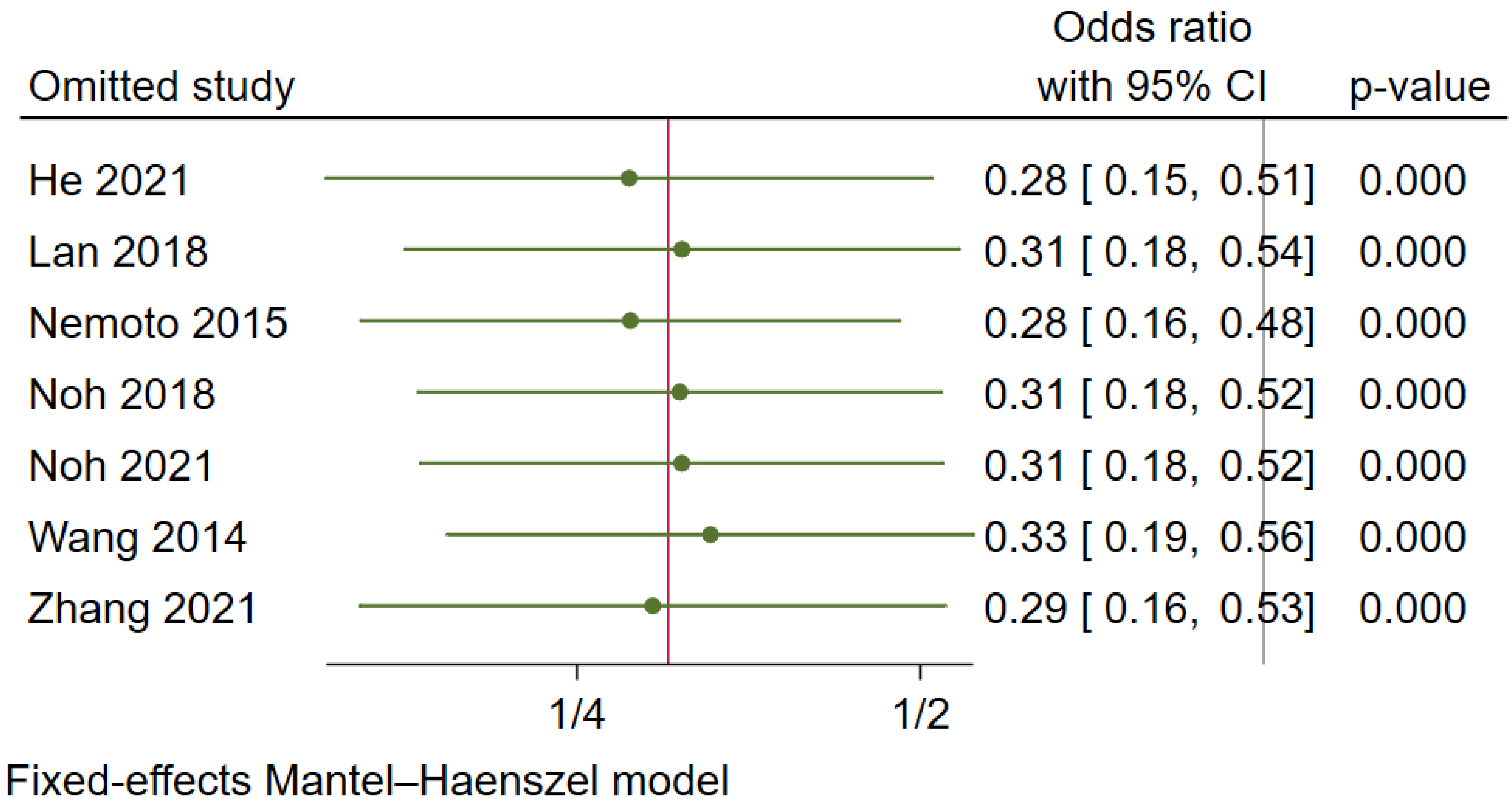

Sensitivity analysis for dysphagia at one month postoperatively

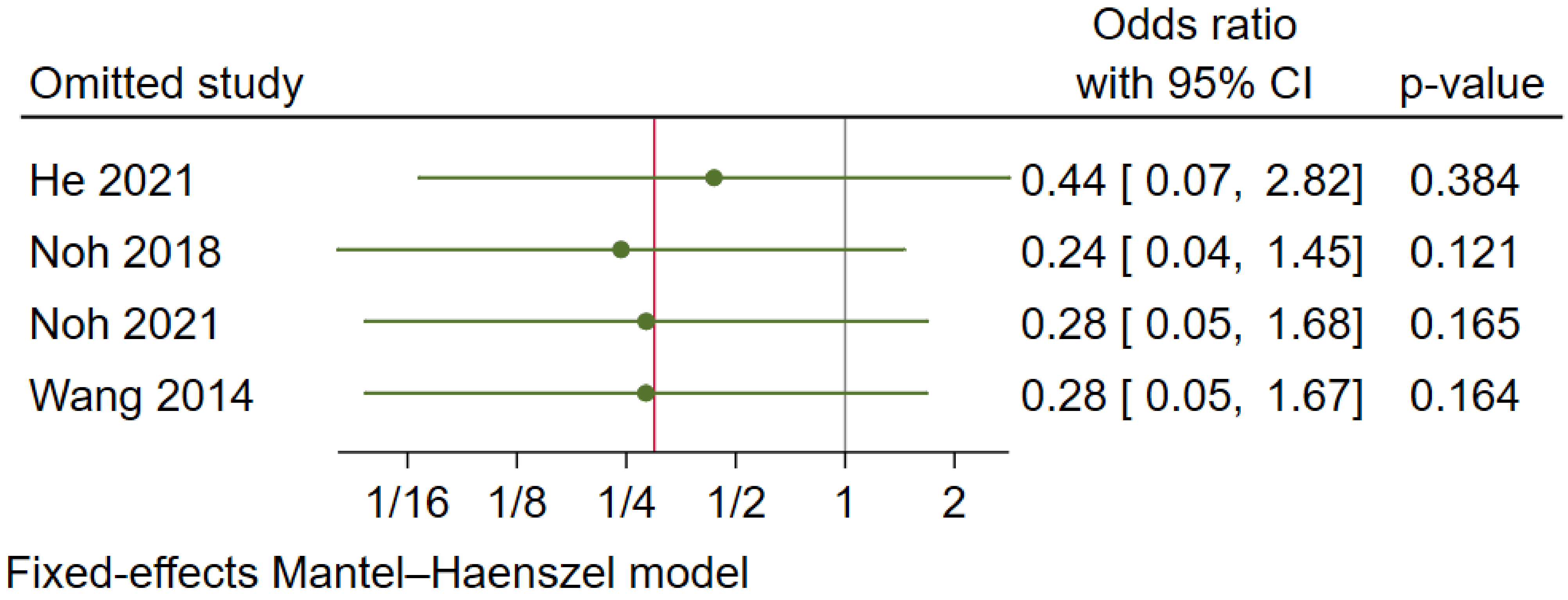

Sensitivity analysis for dysphagia at final follow-up
