# Supplementary material for: Self-locking stand-alone cage versus cage-plate fixation in monosegmental anterior cervical discectomy and fusion with a minimum 2-year follow-up: a systematic review and meta-analysis
Source: J Orthop Surg Res. 2023 Jun 2;18:403. doi: 10.1186/s13018-023-03885-4 (PMC10236847; doi:10.1186/s13018-023-03885-4)
Supplement: Supplementary file 3 — Additional file 3: Meta-regression analysis for the potential sources of heterogeneity. [file 13018_2023_3885_MOESM3_ESM.pdf]

Meta-regression Analysis for the Potential Sources of Heterogeneity

1. Meta-regression analysis of operation time

```
. meta regress year, random(reml)

  Effect-size label: Mean diff.
        Effect size: _meta_es
          Std. err.: _meta_se

Random-effects meta-regression                    Number of obs  =          9
Method: REML                                      Residual heterogeneity:
                                                              tau2 =    21.84
                                                            I2 (%) =    71.15
                                                                H2 =     3.47
                                                     R-squared (%) =     0.00
                                                  Wald chi2(1)   =       0.58
                                                  Prob > chi2    =     0.4479

     _meta_es   Coefficient  Std. err.      z    P>|z|     [95% conf. interval]

         year     -.594109   .7828301    -0.76   0.448    -2.128428    .9402099
        _cons     1187.978   1580.629     0.75   0.452    -1909.999    4285.955

Test of residual homogeneity: Q_res = chi2(7) = 16.55    Prob > Q_res = 0.0205
```

```
. meta regress country, random(reml)

  Effect-size label: Mean diff.
        Effect size: _meta_es
          Std. err.: _meta_se

Random-effects meta-regression                    Number of obs  =          9
Method: REML                                      Residual heterogeneity:
                                                              tau2 =    22.69
                                                            I2 (%) =    63.48
                                                                H2 =     2.74
                                                     R-squared (%) =     0.00
                                                  Wald chi2(1)   =       0.00
                                                  Prob > chi2    =     0.9773

     _meta_es   Coefficient  Std. err.      z    P>|z|     [95% conf. interval]

      country     .1198619   4.210769     0.03   0.977    -8.133093    8.372817
        _cons    -11.69429   3.200655    -3.65   0.000    -17.96746   -5.421121

Test of residual homogeneity: Q_res = chi2(7) = 16.62    Prob > Q_res = 0.0200
```

```
. meta regress study_design, random(reml)

  Effect-size label: Mean diff.
        Effect size: _meta_es
          Std. err.: _meta_se

Random-effects meta-regression                    Number of obs  =          9
Method: REML                                      Residual heterogeneity:
                                                              tau2 =    20.43
                                                            I2 (%) =    71.45
                                                                H2 =     3.50
                                                     R-squared (%) =     0.00
                                                  Wald chi2(1)   =       0.01
                                                  Prob > chi2    =     0.9331

     _meta_es   Coefficient  Std. err.      z    P>|z|     [95% conf. interval]

 study_design    -.5980282   7.124997    -0.08   0.933    -14.56277    13.36671
        _cons    -11.50197   2.105372    -5.46   0.000    -15.62842   -7.375518

Test of residual homogeneity: Q_res = chi2(7) = 16.40    Prob > Q_res = 0.0217

.
```

2. Meta-regression analysis of intraoperative blood loss

```
. meta regress year, random(reml)

  Effect-size label: Mean diff.
        Effect size: _meta_es
          Std. err.: _meta_se

Random-effects meta-regression                      Number of obs  =         9
Method: REML                                        Residual heterogeneity:
                                                                tau2 =   16.19
                                                              I2 (%) =   71.12
                                                                  H2 =    3.46
                                                     R-squared (%) =      2.60
                                                    Wald chi2(1)   =      1.71
                                                    Prob > chi2    =    0.1906

------------------------------------------------------------------------------
    _meta_es | Coefficient  Std. err.      z    P>|z|     [95% conf. interval]
-------------+----------------------------------------------------------------
        year |  -.9225304   .7049128    -1.31   0.191    -2.304134    .4590733
       _cons |   1854.788   1423.358     1.30   0.193    -934.9423    4644.518
------------------------------------------------------------------------------
Test of residual homogeneity: Q_res = chi2(7) = 29.90    Prob > Q_res = 0.0001

.
```

```
. meta regress country, random(reml)

  Effect-size label: Mean diff.
        Effect size: _meta_es
          Std. err.: _meta_se

Random-effects meta-regression                      Number of obs  =         9
Method: REML                                        Residual heterogeneity:
                                                                tau2 =   15.64
                                                              I2 (%) =   68.93
                                                                  H2 =    3.22
                                                     R-squared (%) =      5.92
                                                    Wald chi2(1)   =      1.42
                                                    Prob > chi2    =    0.2329

------------------------------------------------------------------------------
    _meta_es | Coefficient  Std. err.      z    P>|z|     [95% conf. interval]
-------------+----------------------------------------------------------------
     country |   4.708732    3.94738     1.19   0.233     -3.02799    12.44545
       _cons |  -11.26368   3.308204    -3.40   0.001    -17.74764    -4.77972
------------------------------------------------------------------------------
Test of residual homogeneity: Q_res = chi2(7) = 26.63    Prob > Q_res = 0.0004

.
```

```
. meta regress study_design, random(reml)

  Effect-size label: Mean diff.
        Effect size: _meta_es
          Std. err.: _meta_se

Random-effects meta-regression                      Number of obs  =         9
Method: REML                                        Residual heterogeneity:
                                                                tau2 =   18.75
                                                              I2 (%) =   75.56
                                                                  H2 =    4.09
                                                     R-squared (%) =      0.00
                                                    Wald chi2(1)   =      0.55
                                                    Prob > chi2    =    0.4597

------------------------------------------------------------------------------
    _meta_es | Coefficient  Std. err.      z    P>|z|     [95% conf. interval]
-------------+----------------------------------------------------------------
study_design |   6.041305   8.171933     0.74   0.460    -9.975389      22.058
       _cons |  -8.441304   1.977723    -4.27   0.000    -12.31757   -4.565039
------------------------------------------------------------------------------
Test of residual homogeneity: Q_res = chi2(7) = 32.83    Prob > Q_res = 0.0000

.
```

3. Meta-regression analysis of NDI scores

```
. meta regress country

  Effect-size label: Mean diff.
        Effect size: _meta_es
          Std. err.: _meta_se

Random-effects meta-regression                      Number of obs  =          4
Method: REML                                        Residual heterogeneity:
                                                                tau2 =    6.041
                                                              I2 (%) =    94.77
                                                                  H2 =    19.11
                                                       R-squared (%) =     0.00
                                                    Wald chi2(1)   =       0.34
                                                    Prob > chi2    =     0.5581

    _meta_es   Coefficient  Std. err.      z    P>|z|     [95% conf. interval]

     country     -1.808338   3.087799   -0.59   0.558    -7.860313    4.243637
       _cons      1.408337   1.600687    0.88   0.379    -1.728951    4.545625

Test of residual homogeneity: Q_res = chi2(2) =   9.50    Prob > Q_res = 0.0086

.
```

```
. meta regress year

  Effect-size label: Mean diff.
        Effect size: _meta_es
          Std. err.: _meta_se

Random-effects meta-regression                      Number of obs  =          4
Method: REML                                        Residual heterogeneity:
                                                                tau2 =    3.301
                                                              I2 (%) =    78.25
                                                                  H2 =     4.60
                                                       R-squared (%) =     0.00
                                                    Wald chi2(1)   =       1.10
                                                    Prob > chi2    =     0.2939

    _meta_es   Coefficient  Std. err.      z    P>|z|     [95% conf. interval]

        year      .7454145   .7101826    1.05   0.294    -.6465179    2.137347
       _cons     -1505.152   1434.671   -1.05   0.294    -4317.055    1306.751

Test of residual homogeneity: Q_res = chi2(2) =   5.57    Prob > Q_res = 0.0617

.
```
