# Supplementary material for: Self-locking stand-alone cage versus cage-plate fixation in monosegmental anterior cervical discectomy and fusion with a minimum 2-year follow-up: a systematic review and meta-analysis
Source: J Orthop Surg Res. 2023 Jun 2;18:403. doi: 10.1186/s13018-023-03885-4 (PMC10236847; doi:10.1186/s13018-023-03885-4)
Supplement: Supplementary file 4 — Additional file 4: Publication bias assessment. [file 13018_2023_3885_MOESM4_ESM.pdf]

```
. meta bias, egger tdistribution detail

  Effect-size label: Mean diff.
        Effect size: _meta_es
          Std. err.: _meta_se

Random-effects meta-regression                          Number of obs  =         9
Method: REML                                            Residual heterogeneity:
                                                                    tau2 =   5.912
                                                                  I2 (%) =   36.66
                                                                      H2 =    1.58
                                                           R-squared (%) =   61.42
                                                        Model F(1,7)    =     4.08
                                                        Prob > F        =   0.0831

------------------------------------------------------------------------------
    _meta_es | Coefficient  Std. err.      t    P>|t|     [95% conf. interval]
-------------+----------------------------------------------------------------
    _meta_se |  -1.615974   .7997342    -2.02   0.083    -3.507045    .2750974
       _cons |   -5.77399   2.810855    -2.05   0.079     -12.4206    .8726256
------------------------------------------------------------------------------
Test of residual homogeneity: Q_res = chi2(7) = 11.37    Prob > Q_res = 0.1232

Regression-based Egger test for small-study effects
Random-effects model
Method: REML

H0: beta1 = 0; no small-study effects
            beta1 =     -1.62
      SE of beta1 =     0.800
                t =     -2.02
       Prob > |t| =    0.0831

.
```

Publication bias assessment of operation time

```
. meta bias, egger random(reml) tdistribution detail

  Effect-size label: Mean diff.
        Effect size: _meta_es
          Std. err.: _meta_se

Random-effects meta-regression                      Number of obs   =         9
Method: REML                                        Residual heterogeneity:
                                                                tau2 =    11.06
                                                              I2 (%) =    59.27
                                                                  H2 =     2.46
                                                       R-squared (%) =    33.44
                                                    Model F(1,7)     =     3.58
                                                    Prob > F         =   0.1004

------------------------------------------------------------------------------
    _meta_es | Coefficient  Std. err.      t    P>|t|     [95% conf. interval]
-------------+----------------------------------------------------------------
    _meta_se |  -1.552496   .8205591    -1.89   0.100    -3.492809    .3878183
       _cons |  -3.181894   2.886838    -1.10   0.307    -10.00818    3.644394
------------------------------------------------------------------------------
Test of residual homogeneity: Q_res = chi2(7) = 18.99    Prob > Q_res = 0.0082

Regression-based Egger test for small-study effects
Random-effects model
Method: REML

H0: beta1 = 0; no small-study effects
            beta1 =     -1.55
      SE of beta1 =     0.821
                t =     -1.89
      Prob > |t|  =    0.1004

.
```

Publication bias assessment of intraoperative blood loss

```
. meta bias, egger fixed tdistribution detail

  Effect-size label: Mean diff.
        Effect size: _meta_es
          Std. err.: _meta_se

Fixed-effects meta-regression                    Number of obs  =          3
Method: Inverse-variance                         Model F(1,1)   =       2.89
                                                 Prob > F       =     0.3384
```

_meta_es	Coefficient	Std. err.	t	P>|t|	[95% conf.	interval]
_meta_se	-2.858735	1.680864	-1.70	0.338	-24.21614	18.49867
_cons	-.064511	.9185324	-0.07	0.955	-11.73557	11.60655

```
Regression-based Egger test for small-study effects
Fixed-effects model
Method: Inverse-variance

H0: beta1 = 0; no small-study effects
            beta1 =     -2.86
      SE of beta1 =     1.681
                t =     -1.70
       Prob > |t| =    0.3384

.
```

Publication bias assessment of length of hospital stay

```
. meta bias, egger fixed tdistribution detail

  Effect-size label: Mean diff.
        Effect size: _meta_es
          Std. err.: _meta_se

Fixed-effects meta-regression                      Number of obs   =          4
Method: Inverse-variance                           Model F(1,2)    =       2.36
                                                   Prob > F        =     0.2645

------------------------------------------------------------------------------
    _meta_es | Coefficient  Std. err.      t    P>|t|     [95% conf. interval]
-------------+----------------------------------------------------------------
    _meta_se |  -3.528298   2.298095    -1.54   0.264     -13.4162    6.359607
       _cons |   .9129777   .6296767     1.45   0.284    -1.796303    3.622258
------------------------------------------------------------------------------

Regression-based Egger test for small-study effects
Fixed-effects model
Method: Inverse-variance

H0: beta1 = 0; no small-study effects
            beta1 =     -3.53
      SE of beta1 =     2.298
                t =     -1.54
       Prob > |t| =    0.2645

.
```

Publication bias assessment of JOA scores

at the final follow-up

```
. meta bias, egger tdistribution detail

  Effect-size label: Mean diff.
        Effect size: _meta_es
          Std. err.: _meta_se

Random-effects meta-regression                      Number of obs   =         4
Method: REML                                        Residual heterogeneity:
                                                                tau2 =    1.294
                                                              I2 (%) =    80.73
                                                                  H2 =     5.19
                                                       R-squared (%) =     0.00
                                                    Model F(1,2)    =      3.20
                                                    Prob > F        =    0.2156

------------------------------------------------------------------------------
    _meta_es | Coefficient  Std. err.      t    P>|t|     [95% conf. interval]
-------------+----------------------------------------------------------------
    _meta_se |   2.212449   1.236986     1.79   0.216    -3.109873    7.534771
       _cons |  -1.053956   1.101824    -0.96   0.440    -5.794721     3.68681
------------------------------------------------------------------------------
Test of residual homogeneity: Q_res = chi2(2) =  8.98    Prob > Q_res = 0.0112

Regression-based Egger test for small-study effects
Random-effects model
Method: REML

H0: beta1 = 0; no small-study effects
            beta1 =       2.21
      SE of beta1 =      1.237
                t =       1.79
       Prob > |t| =     0.2156

.
```

Publication bias assessment of NDI scores

at the final follow-up

```
. meta bias, egger tdistribution detail

  Effect-size label: Mean diff.
        Effect size: _meta_es
          Std. err.: _meta_se

Fixed-effects meta-regression                     Number of obs  =          4
Method: Inverse-variance                          Model F(1,2)   =       0.02
                                                  Prob > F       =     0.8889
```

_meta_es	Coefficient	Std. err.	t	P>|t|	[95% conf.	interval]
_meta_se	-.1656545	1.047934	-0.16	0.889	-4.674549	4.34324
_cons	-.0904933	.8884819	-0.10	0.928	-3.913322	3.732336

```
Regression-based Egger test for small-study effects
Fixed-effects model
Method: Inverse-variance

H0: beta1 = 0; no small-study effects
            beta1 =     -0.17
      SE of beta1 =     1.048
                t =     -0.16
       Prob > |t| =    0.8889

.
```

Publication bias assessment of cervical Cobb angle at one month postoperatively

```
. meta bias, egger tdistribution detail

  Effect-size label: Mean diff.
        Effect size: _meta_es
          Std. err.: _meta_se

Fixed-effects meta-regression                        Number of obs  =          8
Method: Inverse-variance                             Model F(1,6)   =       4.46
                                                     Prob > F       =     0.0792

------------------------------------------------------------------------------
    _meta_es | Coefficient  Std. err.      t    P>|t|     [95% conf. interval]
-------------+----------------------------------------------------------------
    _meta_se |    1.60379   .7594745     2.11   0.079    -.2545771    3.462157
       _cons |  -2.846377   .7159763    -3.98   0.007    -4.598308   -1.094446
------------------------------------------------------------------------------

Regression-based Egger test for small-study effects
Fixed-effects model
Method: Inverse-variance

H0: beta1 = 0; no small-study effects
            beta1 =      1.60
      SE of beta1 =     0.759
                t =      2.11
       Prob > |t| =    0.0792

.
```

Publication bias assessment of cervical Cobb angle at final follow-up

```
. meta bias, egger tdistribution detail
note: declared Mantel-Haenszel method not supported with meta bias; using inverse-variance method

  Effect-size label: Log odds-ratio
        Effect size: _meta_es
          Std. err.: _meta_se
note: declared Mantel-Haenszel method not supported with meta-regression; using inverse-variance method

Fixed-effects meta-regression                         Number of obs   =          8
Method: Inverse-variance                              Model F(1,6)    =       0.14
                                                      Prob > F        =     0.7247

------------------------------------------------------------------------------
    _meta_es | Coefficient  Std. err.      t    P>|t|     [95% conf. interval]
-------------+----------------------------------------------------------------
    _meta_se |   .2925949   .7927309     0.37   0.725    -1.647148    2.232337
       _cons |  -.7306695   .9085703    -0.80   0.452    -2.953861    1.492522
------------------------------------------------------------------------------

Regression-based Egger test for small-study effects
Fixed-effects model
Method: Inverse-variance

H0: beta1 = 0; no small-study effects
            beta1 =      0.29
      SE of beta1 =     0.793
                t =      0.37
       Prob > |t| =    0.7247

.
```

Publication bias assessment of fusion rate

```
. meta bias, egger tdistribution detail
note: declared Mantel-Haenszel method not supported with meta bias; using inverse-variance method

  Effect-size label: Log odds-ratio
        Effect size: _meta_es
          Std. err.: _meta_se
note: declared Mantel-Haenszel method not supported with meta-regression; using inverse-variance method

Fixed-effects meta-regression                        Number of obs   =         5
Method: Inverse-variance                             Model F(1,3)    =      0.00
                                                     Prob > F        =    0.9493

------------------------------------------------------------------------------
    _meta_es | Coefficient  Std. err.      t    P>|t|     [95% conf. interval]
-------------+----------------------------------------------------------------
    _meta_se |   .0872488   1.262916     0.07   0.949    -3.931914    4.106411
       _cons |   -.214874   .8231266    -0.26   0.811     -2.83443    2.404682
------------------------------------------------------------------------------

Regression-based Egger test for small-study effects
Fixed-effects model
Method: Inverse-variance

H0: beta1 = 0; no small-study effects
            beta1 =      0.09
      SE of beta1 =     1.263
                t =      0.07
       Prob > |t| =    0.9493

.
```

Publication bias assessment of cage subsidence rate

```
. meta bias, egger tdistribution detail
note: declared Mantel–Haenszel method not supported with meta bias; using inverse-variance method

  Effect-size label: Log odds-ratio
        Effect size: _meta_es
          Std. err.: _meta_se
note: declared Mantel–Haenszel method not supported with meta-regression; using inverse-variance method

Fixed-effects meta-regression                       Number of obs   =          4
Method: Inverse-variance                            Model F(1,2)    =       2.56
                                                    Prob > F        =     0.2507

------------------------------------------------------------------------------
    _meta_es | Coefficient  Std. err.      t    P>|t|     [95% conf. interval]
-------------+----------------------------------------------------------------
    _meta_se |    -1.9727   1.232963    -1.60   0.251    -7.277714    3.332314
       _cons |  -.0463553   .6971451    -0.07   0.953    -3.045928    2.953218
------------------------------------------------------------------------------

Regression-based Egger test for small-study effects
Fixed-effects model
Method: Inverse-variance

H0: beta1 = 0; no small-study effects
            beta1 =     -1.97
      SE of beta1 =     1.233
                t =     -1.60
       Prob > |t| =    0.2507

.
```

Publication bias assessment of adjacent segment degeneration rate

```
. meta bias, egger tdistribution detail
note: declared Mantel-Haenszel method not supported with meta bias; using inverse-variance method

  Effect-size label: Log odds-ratio
        Effect size: _meta_es
          Std. err.: _meta_se
note: declared Mantel-Haenszel method not supported with meta-regression; using inverse-variance method

Fixed-effects meta-regression                          Number of obs   =          4
Method: Inverse-variance                               Model F(1,2)    =       0.37
                                                       Prob > F        =     0.6069

------------------------------------------------------------------------------
    _meta_es | Coefficient  Std. err.      t    P>|t|     [95% conf. interval]
-------------+----------------------------------------------------------------
    _meta_se |   8.775759    14.5156     0.60   0.607    -53.67984    71.23136
       _cons |  -15.32286   23.47504    -0.65   0.581    -116.3278    85.68209
------------------------------------------------------------------------------

Regression-based Egger test for small-study effects
Fixed-effects model
Method: Inverse-variance

H0: beta1 = 0; no small-study effects
            beta1 =      8.78
      SE of beta1 =    14.516
                t =      0.60
        Prob > |t| =    0.6069

.
```

Publication bias assessment of dysphagia rate at one month postoperatively

```
. meta bias, egger tdistribution detail
note: declared Mantel-Haenszel method not supported with meta bias; using inverse-variance method

  Effect-size label: Log odds-ratio
        Effect size: _meta_es
          Std. err.: _meta_se
note: declared Mantel-Haenszel method not supported with meta-regression; using inverse-variance method

Fixed-effects meta-regression                         Number of obs  =          7
Method: Inverse-variance                              Model F(1,5)   =       0.61
                                                      Prob > F       =     0.4686

------------------------------------------------------------------------------
    _meta_es | Coefficient  Std. err.      t    P>|t|     [95% conf. interval]
-------------+----------------------------------------------------------------
    _meta_se |   -.916108    1.16875    -0.78   0.469    -3.920476     2.08826
       _cons |  -.5575861   .8183226    -0.68   0.526    -2.661151    1.545979
------------------------------------------------------------------------------

Regression-based Egger test for small-study effects
Fixed-effects model
Method: Inverse-variance

H0: beta1 = 0; no small-study effects
            beta1 =     -0.92
      SE of beta1 =     1.169
                t =     -0.78
       Prob > |t| =    0.4686

.
```

Publication bias assessment of dysphagia rate at final follow-up
